# VS-DCFF: An AI-Based Virtual Sensing Approach for Dual-Target Environmental Parameter Estimation via Deterministic and Copula-Driven Feature Fusion

**DOI:** 10.3390/s26185740

**Published:** 2026-09-09

**Authors:** Muhammad Faizan, Murad Ali Khan, Qazi Waqas Khan, Ji-Eun Kim, Il-yeop Ahn, Do Hyeun Kim

**Affiliations:** 1 Department of Computer Engineering, Jeju National University, Jejusi 63243, Republic of Korea; faizan@stu.jejunu.ac.kr (M.F.);; 2 Autonomous IoT Research Center, Korea Electronics Technology Institute, Seongnam-si 13509, Republic of Korea

**Keywords:** virtual sensing, environmental parameter estimation, machine learning, Gaussian copula, deterministic feature engineering, residual boosting, feature fusion, synthetic data generation, XGBoost, ground-based monitoring, sensor data processing, dual-target prediction, robustness evaluation

## Abstract

Physical sensor deployments in ground-based environmental monitoring networks are frequently constrained by high installation costs, hardware failures, and limited spatial coverage, resulting in incomplete observational datasets and degraded sensing capacity across monitoring stations. Data-driven virtual sensing offers a cost-effective alternative by estimating target environmental parameters through machine learning models trained on correlated sensor measurements, reducing dependency on dense physical infrastructure. This paper presents VS-DCFF, an applied virtual sensing framework for dual-target estimation of near-surface air temperature and relative humidity from ground-based sensor network data. VS-DCFF integrates: (i) a deterministic pipeline applying temporal encoding, rolling-window statistics, and mutual information-based feature selection to capture a linear trend/seasonal component and to select features predictive of the residual signal; (ii) a probabilistic pipeline employing a Gaussian copula model to generate statistically consistent synthetic residual samples preserving inter-variable dependencies; and (iii) an early feature-level fusion strategy feeding a copula-augmented XGBoost residual-boosting stage, whose output is combined with the linear trend component for the final prediction. Under a strict chronological evaluation protocol, VS-DCFF is benchmarked against persistence, linear and ensemble regression baselines, and a same-protocol re-implementation of the statistical core of the VSG-SGL framework, and achieves near-surface air temperature RMSE=0.7791 °C, $R^{2}=0.9735$, and relative humidity RMSE=3.7747%, $R^{2}=0.9701$, outperforming all tested baselines. The framework is further validated through leave-one-station-out spatial generalization, robustness evaluation under simulated sensor faults and target-history loss, copula-variant and synthetic-data fidelity diagnostics, and a lightweight edge-deployment ablation. All findings, including cases where tested extensions such as spatial context features did not yield a robust improvement, are reported transparently. Results indicate that the proposed architecture provides a computationally efficient, extensively validated approach to dual-target environmental virtual sensing under realistic deployment conditions.

## 1. Introduction

Accurate and continuous estimation of environmental variables such as temperature and humidity is essential for a wide range of applications including smart city development, climate analysis, precision agriculture, industrial process control, and public health management [1,2]. Reliable measurement of these variables underpins informed decision-making and system reliability across these domains [3]. Physical sensors remain the dominant measurement modality; however, they are frequently associated with high installation and maintenance costs, hardware failure, sensor drift, calibration requirements, and noise-induced data corruption [4,5]. These limitations degrade system performance and restrict scalability, particularly in large-scale or resource-constrained deployment scenarios where achieving dense and continuous sensor coverage is economically and logistically infeasible.

Virtual sensing has emerged as a promising and cost-effective alternative, estimating target variables through data-driven models trained on correlated, readily available measurements [6,7]. By reducing dependency on physical infrastructure, virtual sensors provide a scalable solution for continuous environmental monitoring without the overhead of dense hardware deployments. Despite their advantages in flexibility and cost efficiency, achieving high prediction accuracy across environmental targets remains challenging. Existing virtual sensor methods predominantly adopt single-pipeline architectures [8] that are insufficient for simultaneously capturing global temporal patterns, local fluctuations, and the stochastic variability inherent in environmental time-series data. Furthermore, real-world environmental datasets are typically characterized by limited sample sizes, high temporal variability, measurement noise, and irregular sampling intervals, all of which collectively hinder model generalization [9].

### 1.1. Motivation and Problem Statement

Recent advances in virtual sensing have explored deep learning architectures, feature engineering pipelines, and hybrid modeling strategies [10,11,12]. While these approaches have demonstrated notable improvements, four persistent limitations motivate the present work. First, most methods rely on a limited and homogeneous feature set, restricting the model’s ability to capture the full spectrum of environmental dynamics [13,14]. Second, the majority of existing models neglect the underlying probabilistic structure and joint dependencies among environmental variables, reducing their effectiveness in stochastic real-world conditions [15]. Third, inadequate handling of temporal dependencies limits model effectiveness in dynamic and non-stationary conditions [9]. Fourth, the absence of synthetic data generation strategies leads to insufficient training data diversity, resulting in poor generalization and overfitting, particularly when real-world observations are scarce or imbalanced [15,16]. A further, related concern is that model architectures are sometimes fixed in advance rather than adapted in response to diagnostic evidence about which components of a signal (e.g., smooth trend versus local residual variation) a given model class is actually well suited to capture. We return to this point in Section 3, where diagnostic findings directly motivated the design of the proposed architecture.

### 1.2. Research Challenges

Building an effective dual-target virtual sensor framework for environmental monitoring involves the following non-trivial challenges: (i) designing a rich and diverse feature space that captures both deterministic temporal structure and the stochastic variability of environmental dynamics; (ii) generating statistically consistent synthetic data that preserves the joint distribution and inter-variable dependencies of the original dataset without distorting real-world statistical properties; (iii) effectively fusing heterogeneous deterministic and probabilistic feature representations into a unified learning-ready dataset; (iv) ensuring robust generalization across both target variables under diverse temporal and seasonal conditions, and across previously unseen monitoring stations; and (v) maintaining computational efficiency suitable for practical deployment in resource-constrained monitoring networks.

### 1.3. Main Contributions

To address the above challenges, this paper presents **VS-DCFF**, an applied virtual sensing framework based on Deterministic and Copula-Driven Feature Fusion for dual-target environmental parameter estimation. We frame VS-DCFF as an applied integrative framework rather than a fundamentally new modeling paradigm: its contribution lies in the specific combination of established components and in a substantially expanded, transparently reported validation protocol. The main contributions of this work are as follows:

- Two-Stage Architecture: A linear trend/seasonal component is paired with a Gaussian copula-augmented XGBoost residual-boosting stage, directing the boosting stage specifically at residual structure a simple regularized model does not already capture.
- Gaussian Copula Residual Augmentation: Generates statistically consistent synthetic residual samples that preserve joint distributions and inter-variable dependencies, validated through marginal-distance, correlation-difference, and tail-dependence diagnostics against alternative copula constructions.
- Early Feature-Level Fusion: Combines deterministic and copula-generated probabilistic features into a unified input space for the residual-boosting stage.
- Dual-Target Evaluation: Validated on 84,582 time-series records from a nine-station environmental monitoring network, covering temperature and humidity prediction, including a joint multi-output variant.
- Strong, Independently Verified Performance: VS-DCFF achieves RMSE=0.7791 °C ($R^{2}=0.9735$) for temperature and RMSE=3.7747% ($R^{2}=0.9701$) for humidity on a strict chronological holdout, outperforming persistence, linear/ensemble regression, and a same-protocol re-implementation of VSG-SGL’s statistical core [8].
- Comprehensive, Transparent Validation: Beyond a single train/test split, the framework is validated through leave-one-station-out spatial generalization, robustness under simulated sensor faults and target-history loss, copula-variant and synthetic-data fidelity diagnostics, and a lightweight edge-deployment ablation, including cases (e.g., spatial context features, Section 6) where a tested extension did not yield a robust improvement.

### 1.4. Paper Organization

The remainder of this paper is organized as follows. Section 2 reviews related work on virtual sensing, feature engineering, and synthetic data generation, and positions VS-DCFF against an expanded set of comparison criteria. Section 3 presents the VS-DCFF methodology, including data preprocessing, the two-stage dual-pipeline architecture, feature fusion, and model training. Section 4 describes the experimental setup, dataset, evaluation protocol, and baseline configurations, including the leakage-free validation-based hyperparameter-selection procedure. Section 5 presents the experimental results across both prediction targets, including the comprehensive baseline comparison, spatial and robustness evaluations, and edge-deployment analysis. Section 6 provides a comprehensive discussion and error-mechanism analysis of the results. Section 7 concludes the paper and outlines specific, actionable future research directions.

## 2. Related Work

Virtual sensing has emerged as a scalable and cost-effective alternative to physical sensors across industrial process monitoring, smart buildings, precision agriculture, and environmental monitoring [6,7,8]. This section reviews representative works organized into four thematic categories and concludes with a comparative analysis identifying the research gaps that motivate the proposed VS-DCFF framework, now assessed against an expanded set of eight design criteria including spatial modeling, extreme-value prediction, and adaptive feature selection.

### 2.1. Deep Learning-Based Virtual Sensors

Deep learning has been widely adopted for virtual sensor modeling due to its capacity to learn complex nonlinear mappings directly from raw data. Zheng et al. [17] proposed a Supervised Hybrid CNN-LSTM network for nonlinear dynamic soft sensing, injecting quality variables into each hidden layer with 1D-to-2D data augmentation, achieving improved performance over LSTM and NARX baselines on industrial fermentation processes. Wang et al. [18] introduced a spatio-temporal consistent and interpretable soft sensor integrating physics-informed latent space loss with a topological alignment block, demonstrating improved generalization across varying operating conditions in coal-fired power plants. Yang et al. [19] proposed a supervised attention-based bidirectional LSTM network combining temporal attention with a moving window quality injection strategy, achieving improved performance over standard LSTM and BiLSTM models for nonlinear industrial quality prediction. Hossain et al. [20] applied Deep Operator Networks as virtual sensors for nuclear reactor digital twins, mapping operational inputs to spatially distributed thermal-hydraulic parameters at considerably faster speeds than conventional CFD simulation. This is notably one of the few reviewed methods to explicitly model spatial field structure, though not extreme-event behavior or adaptive feature selection. Götz et al. [21] introduced a unified multi-task foundation model for virtual sensors, simultaneously predicting diverse sensing targets with cross-task synergy while maintaining computational efficiency for edge deployment. Despite their strong performance, deep learning methods share common limitations: they generally require large labeled datasets, involve considerable computational complexity, offer limited interpretability, and tend to underperform in data-scarce environments [17,20]; none of the reviewed deep learning approaches explicitly targets extreme-value prediction or incorporates adaptive, deployment-time feature reselection.

### 2.2. Machine Learning and Feature Engineering Approaches

Classical machine learning methods combined with systematic feature engineering offer computational efficiency and interpretability advantages over deep learning. Wu et al. [22] developed feature selection-based machine learning for distributed model predictive control, combining filter, wrapper, and embedded selection with reduced-order models for reactor–separator processes, achieving competitive closed-loop performance with improved computational efficiency. Wei et al. [23] proposed adaptive virtual sensors using neighborhood-preserving embedding regression with sparse elastic-net regularization for semiconductor manufacturing, achieving improved performance over global regression methods and preserving local manifold structure in high-dimensional feature spaces. Xu et al. [24] introduced a feature selection method for nonlinear support vector regression via mixed-integer programming, selecting fewer features than standard regularized methods with comparable accuracy and reduced runtime; the selection procedure is performed once and is not revisited under distribution shift, a limitation shared with the present work’s baseline feature-selection design (see Section 6). Curreri et al. [13] provided a systematic review of filter, wrapper, embedded, and causal mutual information-based variable selection methods for data-driven soft sensors, confirming that causal model-inspired approaches tend to generalize better than correlation-based methods. Curreri et al. [14] further surveyed input selection methods for soft sensor design, identifying mutual information as a widely applicable and robust feature ranking criterion across diverse industrial processes. These methods generally assume stationary data distributions and static feature relevance, which may limit their effectiveness in dynamic and non-stationary environmental monitoring scenarios [13,14]; none incorporates spatial correlation across measurement locations or targets extreme-value behavior explicitly.

### 2.3. Building Systems and Environmental Monitoring

Virtual sensing methods applied to building energy systems and environmental monitoring represent the domain most directly relevant to the proposed VS-DCFF framework. Xu et al. [25] proposed a gray-box virtual sensor for room temperature prediction integrating heat transfer physics with multiple linear regression and physical constraints, demonstrating that single-condition training can generalize to multiple HVAC modes with reasonable extrapolation compared to pure black-box models. Xiong et al. [26] developed an in situ calibration approach for HVAC temperature sensor fault-tolerant control using Bayesian inference and principal component analysis with EnergyPlus co-simulation, reporting energy savings after fault-tolerant thermostat control. Li et al. [27] introduced a hybrid feature selection framework for indoor temperature prediction in large-space buildings combining transfer entropy with a gated recurrent unit wrapper and lifecycle cost analysis, identifying cost-efficient feature subsets under different prediction horizons. Khan et al. [8] proposed VSG-SGL, combining sparse Gaussian Process Regression and Bayesian Ridge Regression with generative augmentation, anomaly detection, and kernel density estimation validation for meteorological variable prediction across South Korean monitoring stations, the same geographic context as the dataset used in this study. We note that VSG-SGL validates virtual sensor quality primarily through downstream sequence-model (BiLSTM/BiGRU) forecasting error on normalized data rather than direct regression metrics on a chronological holdout in physical units; in Section 4 we re-implement its statistical estimation core under the present study’s evaluation protocol to enable a direct, same-unit comparison. Ahmadnia et al. [28] addressed building sensor coverage through dual integer linear programming models with a leader–follower bi-level approach, achieving a cost–coverage balance for smart building air quality sensor placement. This is a spatial-coverage optimization perspective that is complementary to, but distinct from, spatial feature modeling for prediction accuracy. While these works demonstrate the viability of virtual sensing in environmental and building contexts, none integrates deterministic feature engineering with probabilistic synthetic data generation through a unified feature-level fusion strategy, and none explicitly addresses extreme-value prediction or adaptive, deployment-time feature selection.

### 2.4. Data Augmentation and Synthetic Generation

Data augmentation and synthetic data generation have gained increasing attention as strategies to address training data scarcity and improve model generalization in sensor applications. El Kojok et al. [15] proposed a VAE-GAN augmentation pipeline for medical image classification, achieving lower Fréchet Inception Distance than alternative augmentation methods and improving classification accuracy from 73% to 89%, demonstrating that generative augmentation strategies can contribute to improved generalization. Zhao et al. [16] developed a Heterogeneous Temporal Graph Neural Network for virtual sensing in complex systems, treating multi-modal sensor nodes as distinct graph node types with exogenous variable context integration, achieving improved performance over compared methods for bearing load and bridge live load prediction under varying operating conditions. This method explicitly incorporates both spatial (graph-structured) relationships and, through its load-prediction application, exposure to extreme operating regimes, making it the most feature-complete reviewed method on these two criteria, albeit without addressing adaptive feature selection. Lee et al. [10] reviewed emerging trends in machine learning-enhanced sensor technologies, highlighting that data augmentation and synthetic generation strategies are increasingly relevant for robust sensor modeling in data-scarce deployments. Despite their contributions, existing augmentation approaches either require complex generative architectures or do not preserve the joint statistical dependencies among multiple environmental variables, which may limit their applicability to dual-target monitoring scenarios [15,16].

### 2.5. Research Gap and Positioning

A review of the discussed works reveals four persistent limitations that collectively motivate the proposed VS-DCFF framework: (i) limited feature diversity from homogeneous single-pipeline representations [13,14]; (ii) absence of copula-based probabilistic synthesis that preserves joint inter-variable dependencies [15,16]; (iii) inadequate handling of temporal non-stationarity, static (non-adaptive) feature selection, and data scarcity in environmental time-series monitoring [9]; and (iv) lack of a unified early feature-level fusion strategy combining deterministic and probabilistic representations for dual-target prediction [8]. We additionally observe that spatial correlation across monitoring locations and explicit treatment of extreme-value behavior are addressed by at most one or two of the reviewed methods each (notably DeepONet [20] and HTGNN [16] for spatial structure), and that no reviewed method incorporates adaptive, deployment-time feature reselection. No existing approach in the reviewed literature simultaneously addresses all eight criteria for dual-target environmental prediction covering both temperature and humidity within a single scalable framework, nor, as discussed in Section 6, does the present work, which we report honestly rather than claim otherwise. In contrast to the most closely related work VSG-SGL [8], which employs Bayesian Ridge Regression and Gaussian Process Regression but does not incorporate a copula-based synthetic generation strategy or a unified feature-level fusion module, the proposed VS-DCFF framework integrates a Gaussian copula pipeline with deterministic feature engineering through early fusion, providing richer synthetic diversity and more informative joint feature representations. Section 4 additionally provides a direct, same-protocol empirical comparison against a re-implementation of VSG-SGL’s statistical core. Table 1 summarizes the positioning of VS-DCFF relative to key reviewed approaches across eight design criteria.

We report VS-DCFF’s own positioning candidly rather than assertively. Dual-target capability is marked partially addressed (∘), reflecting that the primary configuration trains per-target models, with a joint multi-output variant evaluated as a secondary configuration (Section 5). Spatial modeling is likewise marked partially addressed (∘): we conducted two independent investigations of spatial context features (one using an unweighted neighbor-average with verified station identity, one using true distance-weighted features from supplementary coordinate data), which produced inconsistent results across dataset constructions and were therefore not incorporated into the final architecture (Section 6). Extreme-value prediction and adaptive feature selection are marked not addressed (×) and are identified as concrete future work (Section 7).

## 3. Methodology

This section presents the methodology of the proposed VS-DCFF framework. A comprehensive baseline comparison, reported in Section 5, showed that a single-stage deterministic-plus-copula-fusion architecture did not consistently outperform simple regularized regression on the same engineered features. Motivated by this diagnostic finding, the framework is structured as a two-stage architecture: a linear trend/seasonal component fit directly on the deterministic feature set, followed by a Gaussian copula-augmented XGBoost residual-boosting stage that models the structure remaining in the linear model’s residuals. This design directs the gradient-boosted component specifically at the part of the signal where it provides genuine, empirically verified benefit, rather than competing directly with a simpler model on structure the simpler model already captures well.

### 3.1. Framework Overview

**Figure 1:**
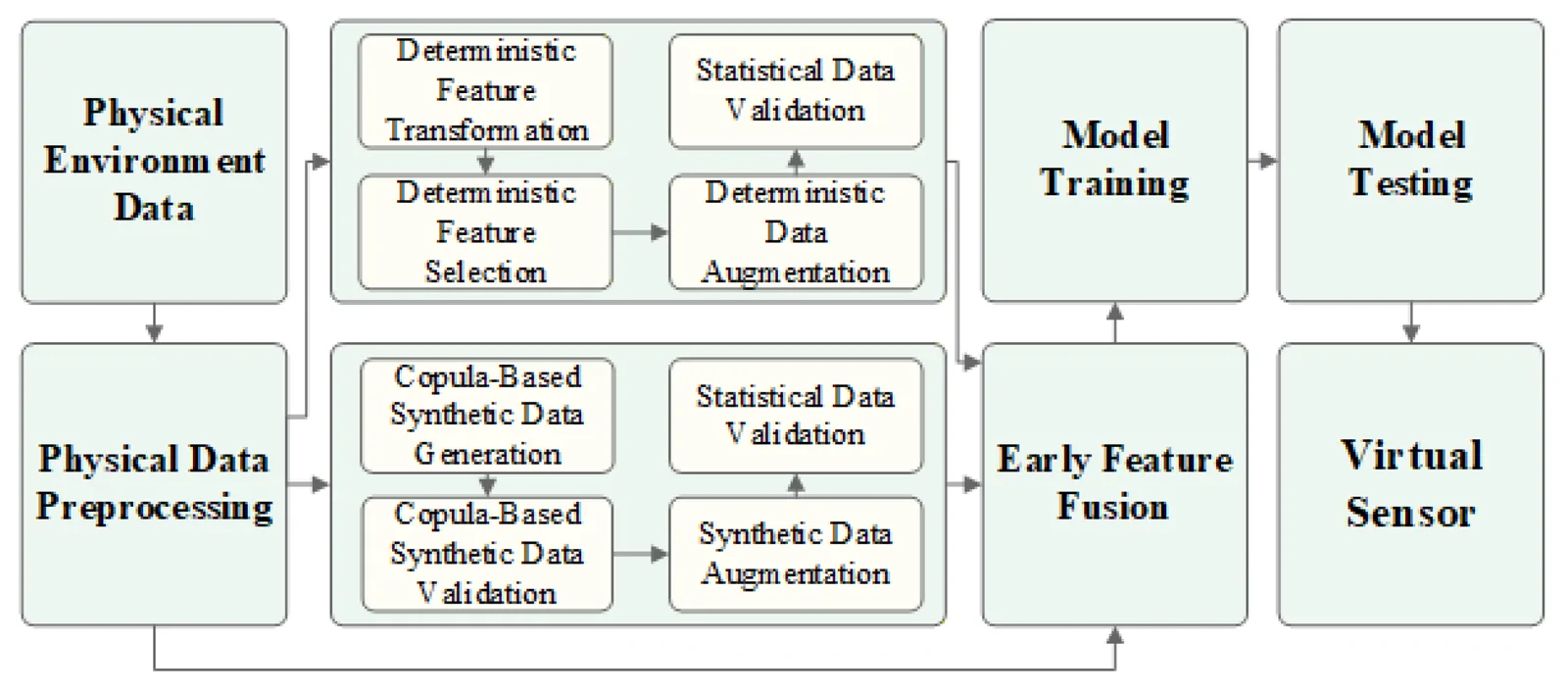
General overview of the proposed VS-DCFF framework showing the end-to-end two-stage pipeline, from data collection through the linear trend component, residual-boosting stage, and final evaluation.

**Figure 2:**
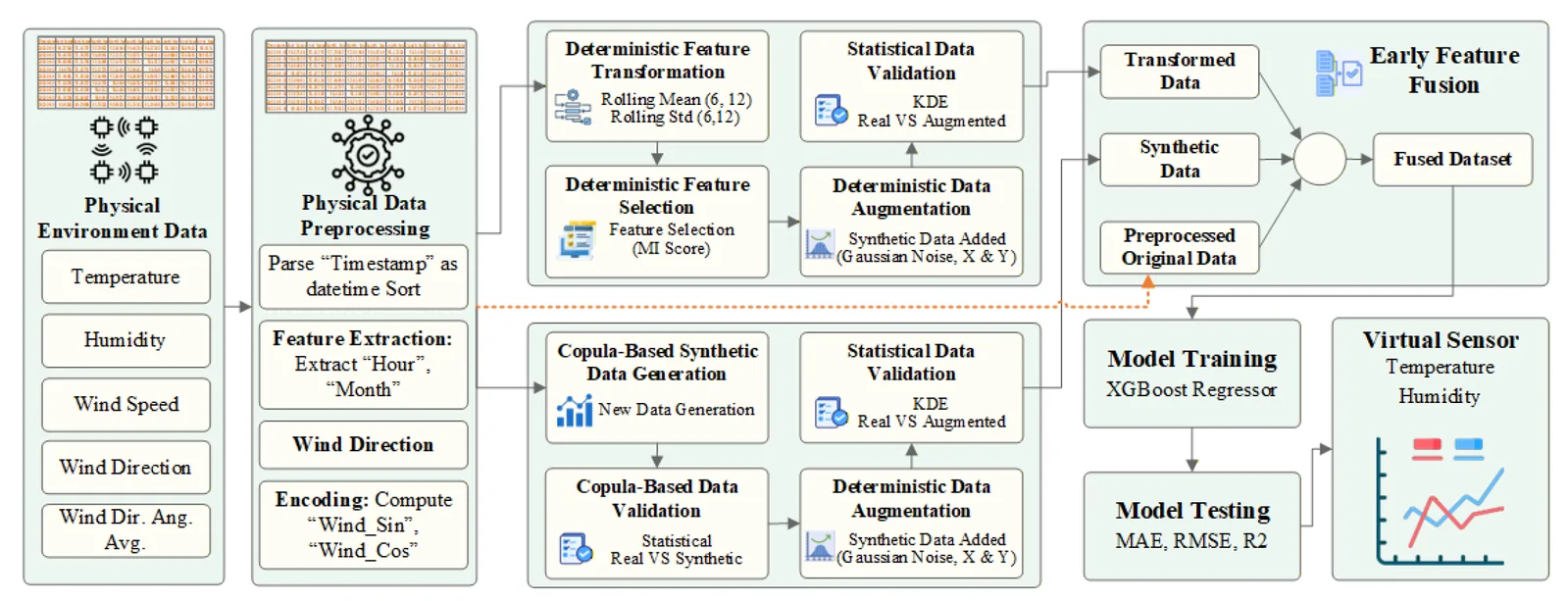
Detailed architecture of VS-DCFF showing the linear trend component, the deterministic residual-feature pipeline (Pipeline A), the cross-sensor BRR/GPR estimation stage, the probabilistic Gaussian copula residual-synthesis pipeline (Pipeline B), the early feature-level fusion module, and the residual-boosting XGBoost model whose output is combined with the linear trend prediction.

The VS-DCFF pipeline is organized into seven sequential stages: (i) data preprocessing, including station-aware temporal structuring; (ii) a linear trend/seasonal component fit on the deterministic feature set; (iii) deterministic residual-feature transformation and mutual-information-based selection, computed against the linear model’s residual rather than the raw target; (iv) cross-sensor statistical estimation via Bayesian Ridge Regression (BRR) and sparse Gaussian Process Regression (GPR), providing auxiliary predictive columns for the residual-boosting stage; (v) probabilistic synthetic residual generation and validation via a Gaussian copula; (vi) early feature-level fusion and Gaussian noise augmentation; and (vii) residual-boosting model training and evaluation, with the final prediction obtained by adding the residual booster’s output to the linear trend prediction. Figure 1 illustrates the general end-to-end framework overview, and Figure 2 presents the detailed architecture; both figures illustrate the two-stage linear-trend-plus-residual-boosting structure described below.

The linear trend component $g_{\mathrm{lin}}$ is fit on the full deterministic feature set and captures the smooth seasonal and diurnal structure that dominates both target variables. The *Deterministic Pipeline A* performs temporal encoding, cyclic variable transformation, and rolling statistical feature extraction, with mutual-information-based selection computed against the linear model’s *residual* $r_{t}=y_{t}-g_{\mathrm{lin}}\left(x_{t}\right)$ rather than the raw target, so that selected features are specifically informative about the structure the linear component has not already captured. A *cross-sensor estimation stage* fits BRR and sparse GPR models predicting each target from the other target and wind-related variables only (no target-history information), providing two additional auxiliary columns to the residual-boosting feature space. The *Probabilistic Pipeline B* operates on this residual-augmented feature space, employing a Gaussian copula model fitted jointly over the selected features, the auxiliary BRR/GPR columns, and the residual target, to generate statistically consistent synthetic observations. Both pipelines feed into an *Early Fusion* module (Section 3.7) that concatenates real and synthetic observations into a unified dataset for residual-boosting model training. Table 2 summarizes the principal mathematical symbols used throughout this section.

### 3.2. Data Preprocessing

The preprocessing pipeline ensures temporal consistency, data quality, and foundational feature extraction across both prediction targets, with explicit attention to station identity throughout.

#### 3.2.1. Temporal Structuring and Missing Value Handling

Raw timestamps are parsed to date/time format. All rolling-window, lag, and temporal-encoding features described in Section 3.4 are computed *group-wise by station*: records are first grouped by station identifier, and only then sorted chronologically and processed within each group, so that no rolling window or lag feature ever spans observations from two different stations:

(1) $$t_{i}=\mathrm{datetime}\left({\mathrm{raw}\text{\_}\mathrm{timestamp}}_{i}\right),\;D_{\mathrm{sorted}}=\underset{s\in S}{⋃}\mathrm{sort}\left(D_{s},\;\mathrm{by}\;t\right),$$

where S denotes the set of station identifiers and $D_{s}$ denotes the subset of records belonging to station *s*.

Records containing missing values are removed:

(2) $$D_{\mathrm{clean}}=\left\{\left(x_{t},y_{t}\right)\in D_{\mathrm{sorted}}\;|\;x_{t}\neq \mathrm{NaN}\;\wedge \;y_{t}\neq \mathrm{NaN}\right\}.$$

Every removed record is fully empty (timestamp and all sensor fields simultaneously null), consistent with collection-period truncation or communication outage at the affected station rather than scattered per-field sensor faults (missingness ranges from 8.9% to 33.0% by station and is heavily concentrated toward the end of each station’s individual collection window; full characterization is reported in Section 4). Because no valid timestamp is available for these records, no temporal anchor exists for interpolation or model-based imputation, making complete-case deletion the only coherent option for this specific missingness pattern.

Temporal features are extracted to capture periodic and seasonal behavior:

(3) $${\mathrm{Hour}}_{t}=\mathrm{hour}\left(t_{i}\right),\;{\mathrm{Month}}_{t}=\mathrm{month}\left(t_{i}\right),\;{\mathrm{Day}}_{t}=\mathrm{day}\left(t_{i}\right),\;{\mathrm{DoW}}_{t}=\mathrm{dayofweek}\left(t_{i}\right).$$

#### 3.2.2. Wind Direction Encoding

Wind direction is a circular variable requiring trigonometric encoding to eliminate angular discontinuity at the $0^{\circ }$ to $360^{\circ }$ boundary:

(4) $$\theta =\frac{2\pi \cdot \mathrm{wind}\text{\_}\mathrm{dir}}{360},\;\mathrm{wind}\text{\_}\sin=\sin\left(\theta \right),\;\mathrm{wind}\text{\_}\cos=\cos\left(\theta \right).$$

### 3.3. Linear Trend Component

The linear trend component is an ordinary least squares regression fit on the full deterministic feature set $x_{t}$ (temporal encodings, rolling statistics, and lag features, per Section 3.4):

(5) $$g_{\mathrm{lin}}\left(x_{t}\right)=w^{⊤}x_{t}+b,\;\left(w^{*},b^{*}\right)=\arg\min_{w,b}\sum_{t=1}^{N_{\mathrm{train}}}{\left(y_{t}-w^{⊤}x_{t}-b\right)}^{2},$$

fit exclusively on the training partition. This component captures the dominant smooth seasonal and diurnal structure of both targets; the residual

(6) $$r_{t}=y_{t}-g_{\mathrm{lin}}\left(x_{t}\right)$$

is the target for all subsequent stages of the pipeline. To prevent target leakage, all target-derived rolling-statistic and lag features entering $x_{t}$ including those consumed by the linear trend component $g_{\mathrm{lin}}$ are computed exclusively from strictly past observations $\left\{z_{t-1},\ldots ,z_{t-k}\right\}$; the contemporaneous value $z_{t}$ is never included in any rolling window or lag feature derived from the target itself (Section 3.4). This applies uniformly to the full deterministic feature set used to fit $g_{\mathrm{lin}}$, not only to the residual-stage features of Pipeline A. We note that multi-output ordinary least squares regression decomposes into independent per-target solutions of Equation (5); a jointly fit linear component is therefore mathematically equivalent to per-target fitting, and the meaningful test of joint dual-target modeling lies in the residual-boosting stage (Section 3.9).

### 3.4. Deterministic Residual-Feature Transformation and Selection: Pipeline A

Pipeline A extracts structured temporal features from the real dataset and applies mutual information-based selection, computed against the residual target $r_{t}$, to retain the most informative subset for the residual-boosting stage.

#### 3.4.1. Rolling Statistics

Rolling mean and standard deviation are computed group-wise by station over window size *k* for each input variable *z*:

(7) $$\mu_{t}^{\left(k\right)}=\frac{1}{k}\sum_{i=0}^{k-1}z_{t-i},$$

(8) $$\sigma_{t}^{\left(k\right)}=\sqrt{\frac{1}{k}\sum_{i=0}^{k-1}{\left(z_{t-i}-\mu_{t}^{\left(k\right)}\right)}^{2}}.$$

Window sizes K = 6, 12 samples (1 h and 2 h at the 10-minute sampling interval) are used to capture short-term, sub-daily dynamics. Lag features $z_{t-1}$, $z_{t-2}$, and $z_{t-3}$ are additionally appended to encode short-term historical context; both the rolling-statistic and lag features of the target variable itself are available as candidate inputs, corresponding to a sensor-replacement/gap-filling deployment mode in which a station’s own recent history remains available (Section 4). For target-derived variables z∈{temperature,humidity}, the rolling window in Equations (7) and (8) and the lag features $z_{t-1}$, $z_{t-2}$, $z_{t-3}$ are computed using only observations strictly prior to *t*; the current-timestep value $z_{t}$ is excluded from every rolling and lag feature derived from the target, preventing direct leakage of $y_{t}$ into its own predictors. Non-target (auxiliary sensor) rolling features are unaffected, as no leakage pathway exists for variables other than the prediction target itself.

#### 3.4.2. Feature Selection via Mutual Information

Mutual information ranks features by their non-redundant informational contribution to the *residual* target:

(9) $$I\left(x_{i};\;r\right)=\sum_{x_{i},\;r}p\left(x_{i},r\right)\log\left[\frac{p(x_{i},r)}{p\left(x_{i}\right)\;p\left(r\right)}\right],$$

estimated using a histogram-based approach with bin counts determined by the Freedman–Diaconis rule applied independently to each feature. Histogram-based MI estimators are known to exhibit finite-sample bias that decreases as bin width is chosen adaptively to the local data density, in contrast to *k*-nearest-neighbor-based estimators (e.g., the Kraskov–Stögbauer–Grassberger estimator), which trade this bias for higher variance at small sample sizes; given the sample sizes available per station in this study, the histogram-based estimator was preferred for its lower computational cost at comparable bias. Physically, high MI scores for rolling-statistic features indicate that local volatility or trend information not captured by the linear component remains informative about the residual, while high MI scores for temporal-encoding features (e.g., hour, day) indicate residual structure tied to within-day or within-month effects not fully absorbed by the linear model’s coefficients. A top-*K* selection strategy (default K=8) retains the most informative features per target, forming the deterministic residual-feature matrix $X^{\det}$.

### 3.5. Cross-Sensor Statistical Estimation

To provide an auxiliary predictive signal independent of the target’s own history, two statistical estimators are fitted using only the other target variable and wind-related inputs (windspeedmax, wind_sin, wind_cos) as predictors, following the cross-sensor design of VSG-SGL [8]:

Bayesian Ridge Regression (BRR). A Bayesian linear model with a Gaussian prior on the regression weights, providing a point prediction $h_{\mathrm{BRR}}\left(x_{t}^{\mathrm{cross}}\right)$ and closed-form posterior uncertainty.

Sparse Gaussian Process Regression (GPR). A Gaussian Process with an RBF-plus-white-noise kernel, fitted on a fixed-size random subsample (400 points) of the training set to control the $O(N^{3})$ computational cost of exact GPR, providing prediction $h_{\mathrm{GPR}}\left(x_{t}^{\mathrm{cross}}\right)$.

Both estimators’ predictions, ${\mathrm{syn}\text{\_}\mathrm{brr}}_{t}=h_{\mathrm{BRR}}\left(x_{t}^{\mathrm{cross}}\right)$ and ${\mathrm{syn}\text{\_}\mathrm{gpr}}_{t}=h_{\mathrm{GPR}}\left(x_{t}^{\mathrm{cross}}\right)$, are appended as two additional columns to the residual-boosting feature space $X^{\det}$, giving the pre-fusion feature matrix $X^{b}$ used as input to Pipeline B.

### 3.6. Probabilistic Synthetic Residual Generation: Pipeline B

Pipeline B employs a Gaussian copula model to generate synthetic *residual-stage* observations, comprising the selected deterministic features, the cross-sensor BRR/GPR columns, and the residual target jointly, that preserve the joint distribution and inter-variable dependencies of the real training data, producing the synthetic dataset $D_{2}$.

#### 3.6.1. Sklar’s Theorem and the Gaussian Copula

For a *d*-dimensional random vector $V=(V_{1},\ldots ,V_{d})$ with joint cumulative distribution function *H* and marginal CDFs $F_{1},\ldots ,F_{d}$, Sklar’s theorem guarantees the existence of a copula function *C* such that

(10) $$H\left(v_{1},\ldots ,v_{d}\right)=C\left(F_{1}\left(v_{1}\right),\ldots ,F_{d}\left(v_{d}\right)\right),$$

and, when each $F_{j}$ is continuous, *C* is unique. Equation (10) formalizes the separation of marginal behavior (captured by $F_{1},\ldots ,F_{d}$) from dependence structure (captured by *C*) that motivates the copula approach: marginal distributions of individual variables can be modeled or estimated independently of how those variables co-vary. The Gaussian copula specifies *C* as the joint CDF of a multivariate normal distribution with zero mean, unit variances, and correlation matrix Σ, applied to the probit-transformed marginals:

(11) $$C_{\Sigma }\left(u_{1},\ldots ,u_{d}\right)=\Phi_{\Sigma }\left(\Phi^{-1}\left(u_{1}\right),\ldots ,\Phi^{-1}\left(u_{d}\right)\right),$$

with density

(12) $$c_{\Sigma }\left(u_{1},\ldots ,u_{d}\right)=\frac{1}{\sqrt{\det\Sigma }}\exp\left(-\frac{1}{2}z^{⊤}\left(\Sigma^{-1}-I\right)z\right),\;z_{j}=\Phi^{-1}\left(u_{j}\right).$$

The elliptical dependence structure implied by Equation (12) is symmetric and cannot represent asymmetric tail dependence; we quantify the practical consequence of this limitation directly via tail-dependence diagnostics in Section 5, rather than relying on this theoretical observation alone.

The four-step generation procedure operationalizes Equations (10)–(12):

Step 1: Rank Transformation.

Each variable $x^{\left(j\right)}$ (drawn from $X^{b}$ and *r* jointly) is transformed to a uniform marginal using the empirical rank transformation

(13) $$u_{i}^{\left(j\right)}=\frac{\mathrm{rank}(x_{i}^{\left(j\right)})}{N+1},\;j=1,\ldots ,d,$$

computed exclusively on the training partition, where *N* is the number of training observations. The empirical rank transformation is a consistent estimator of the true probability integral transform $F_{j}\left(x^{\left(j\right)}\right)$ as N→∞, by the Glivenko–Cantelli theorem applied to the empirical CDF.

Step 2: Gaussian Transformation.

(14) $$z_{i}^{\left(j\right)}=\Phi^{-1}\left(u_{i}^{\left(j\right)}\right),$$

yielding a multivariate Gaussian representation $z_{i}\in R^{d}$.

Step 3: Covariance Estimation and Sampling.

(15) $$\Sigma =\mathrm{Cov}\left(Z\right),\;{\tilde{z}}_{i}∼N\left(0,\;\Sigma \right),\;i=1,\ldots ,\left\lfloor \phi N\right\rfloor ,$$

where ϕ=0.25 is the copula synthetic-sample fraction (Section 4), so that ⌊ϕN⌋ synthetic observations are drawn.

Step 4: Back-Transformation.

(16) $${\tilde{x}}_{i}^{\left(j\right)}=F_{j}^{-1}\left(\Phi ({\tilde{z}}_{i}^{\left(j\right)})\right),$$

using the empirical quantile function $F_{j}^{-1}$ of the training data, ensuring that synthetic samples preserve marginal distributions and joint dependencies of the real data. Because Equation (15) samples *d*-dimensional vectors jointly, each synthetic observation carries its own synthetic value for the residual-target dimension: synthetic feature rows are never paired with real target labels, and the covariance matrix Σ is estimated exclusively from the training partition, so no synthetic sample is ever informed by or placed in the test set.

#### 3.6.2. Synthetic Data Validation

Generated synthetic data is validated through: (i) Kolmogorov–Smirnov statistics and Wasserstein-1 distances between real and synthetic marginals for every variable; (ii) correlation-matrix difference analysis (maximum absolute entry-wise difference and Frobenius norm) confirming preservation of inter-variable dependency structure; (iii) pairwise real-vs-synthetic scatter overlays; and (iv) empirical upper- and lower-tail dependence coefficients at the 95th and 99th percentiles, directly quantifying the practical impact of the elliptical-dependence assumption noted above. Quantitative results of this validation are reported in Section 5.

#### 3.6.3. Comparison with Alternative Copula Constructions

The Gaussian copula was compared against an R-vine copula (permitting Gaussian, Clayton, Gumbel, and Frank pair-copulas selected per edge, capturing asymmetric tail dependence) and a nonparametric resampling approach (bootstrap resampling of real rows with per-variable Silverman-bandwidth jitter). Full comparative results, including the accuracy–computational-cost trade-off that motivates retaining the Gaussian copula as the default, are reported in Section 5.

### 3.7. Early Feature-Level Fusion and Augmentation

The real residual-stage observations $\left(X^{b},r\right)$ and the copula-synthetic observations $\left({\tilde{X}}^{b},\tilde{r}\right)$ are combined by row-wise concatenation into a unified fused dataset:

(17) $$X^{\mathrm{fused}}=\left[\begin{array}{c} X^{b}\\ {\tilde{X}}^{b} \end{array}\right],\;r^{\mathrm{fused}}=\left[\begin{array}{c} r\\ \tilde{r} \end{array}\right],$$

i.e., a feature-space direct sum of the real and synthetic observation sets sharing an identical column structure (selected deterministic features plus the two cross-sensor BRR/GPR columns). Fusion here combines *real and synthetic rows* within a single, unified residual-stage feature space, rather than concatenating separately engineered feature blocks column-wise.

Gaussian noise augmentation is then applied to $\left(X^{\mathrm{fused}},r^{\mathrm{fused}}\right)$, doubling the dataset by appending a jittered copy:

(18) $${\tilde{X}}^{\mathrm{aug}}=X^{\mathrm{fused}}+\varepsilon_{X},\;{\tilde{r}}^{\mathrm{aug}}=r^{\mathrm{fused}}+\varepsilon_{r},\;\varepsilon_{X},\varepsilon_{r}∼N\left(0,\;\sigma_{\mathrm{aug}}^{2}\right),\;\sigma_{\mathrm{aug}}=0.01.$$

Applied at this residual-boosting stage, additive input noise is a classical form of Tikhonov-type regularization: training on x+ε is equivalent, in expectation, to minimizing a penalized empirical risk objective that discourages the fitted function from relying on high-frequency, low-margin decision boundaries, constraining the effective complexity of the residual booster. We revisit the magnitude of this effect empirically in Section 5 and Section 6, since the residual target’s scale differs substantially from the raw target’s scale.

### 3.8. Residual-Boosting Model Training and Evaluation

#### 3.8.1. Model Formulation

The residual booster is an additive ensemble of *M* decision trees trained on the fused, augmented residual-stage dataset:

(19) $${\hat{r}}_{t}=\sum_{m=1}^{M}f_{m}\left(x_{t}^{\mathrm{fused}}\right),$$

minimizing the regularized objective

(20) $$L=\sum_{t}\ell \left({\hat{r}}_{t},\;r_{t}\right)+\sum_{m=1}^{M}\Omega \left(f_{m}\right),\;\Omega \left(f_{m}\right)=\gamma T_{m}+\frac{1}{2}\lambda_{\mathrm{xgb}}{∥w_{m}∥}^{2},$$

where $T_{m}$ is the number of leaves and $\lambda_{\mathrm{xgb}}$ controls $L_{2}$ regularization. The final prediction combines the linear trend and residual-boosting stages:

(21) $${\hat{y}}_{t}=g_{\mathrm{lin}}\left(x_{t}\right)+{\hat{r}}_{t}.$$

We do not derive a closed-form generalization bound for Equation (19): gradient-boosted tree ensembles do not admit the bounds available for linear or kernel methods without assumptions the architecture does not satisfy. In place of a formal bound, the regularization argument of Section 3.7 and the empirical ablation, sensitivity, and robustness studies of Section 5 and Section 6 provide direct, measured evidence of the model’s stability and generalization behavior.

#### 3.8.2. Hyperparameter Selection

Hyperparameters (D∈{4,5,6,7}, η∈{0.05,0.03,0.02}, M=300) are selected via mean validation RMSE across k=5 expanding TimeSeriesSplit folds, computed *exclusively on real, chronologically ordered training rows* $\left(X^{b},r\right)$, prior to copula synthesis and noise augmentation. This exclusion is necessary because copula-synthetic and noise-augmented rows are generated from statistics of the entire training partition and, if included in the cross-validation folds themselves, can leak information across a fold boundary, inflating validation performance for configurations that overfit this leakage rather than generalizing genuinely. The selected configuration is then refitted on the full fused, augmented dataset $\left({\tilde{X}}^{\mathrm{aug}},{\tilde{r}}^{\mathrm{aug}}\right)$, and the held-out chronological test set is used exactly once, for final evaluation only. Hyperparameters are selected by mean validation RMSE rather than minimum training RMSE, since the latter would favor configurations that fit the training set most closely rather than those that generalize best. Critically, all supervised preprocessing internal to each cross-validation fold fitting the linear trend component (Section 3.3), computing the residual target, performing mutual-information feature selection against that fold’s own residual (Section 3.4), and fitting the cross-sensor BRR/GPR estimators (Section 3.5) is refitted independently within each fold’s training subset and then applied to that fold’s validation subset; no fold’s validation rows influence their own residualization, feature selection, or auxiliary feature generation. This nesting applies only to the hyperparameter-selection loop (Algorithm 3); the final model is refitted once on the full training partition after the best configuration is selected. Table 3 reports fold-wise validation RMSE, MAE, and $R^{2}$ (mean and standard deviation across the five expanding folds) for both targets.

#### 3.8.3. Evaluation Metrics

Model performance is assessed using three standard regression metrics:

(22) $$\mathrm{RMSE}=\sqrt{\frac{1}{N}\sum_{t=1}^{N}{\left({\hat{y}}_{t}-y_{t}\right)}^{2}},$$

(23) $$\mathrm{MAE}=\frac{1}{N}\sum_{t=1}^{N}\left|{\hat{y}}_{t}-y_{t}\right|,$$

(24) $$R^{2}=1-\frac{\sum_{t}{\left({\hat{y}}_{t}-y_{t}\right)}^{2}}{\sum_{t}{\left(y_{t}-\bar{y}\right)}^{2}}.$$

All metrics are computed on final predictions ${\hat{y}}_{t}$ (Equation (21)), i.e., on the original target scale, not the residual scale.

### 3.9. Multi-Output Variant

Because the linear trend component (Section 3.3) is mathematically equivalent whether fitted jointly or per-target, the meaningful test of joint dual-target modeling lies in the residual-boosting stage. We additionally implement and evaluate a joint variant in which a single multi-output XGBoost model (using native multi-output tree construction) predicts both targets’ residuals from a shared, unioned feature space, rather than training two independent per-target boosters. Comparative results are reported in Section 5; the per-target design is retained as the primary configuration, with the joint variant reported as a secondary, single-model alternative.

### 3.10. Algorithms

This section presents the algorithmic specification of the VS-DCFF framework, decomposed into three self-contained algorithms covering the linear trend and deterministic residual-feature pipeline, Gaussian copula residual synthesis, and residual-boosting training with leakage-free hyperparameter selection.

#### 3.10.1. Algorithm 1: Linear Trend Fitting and Deterministic Residual-Feature Selection

Algorithm 1 presents the linear trend fitting step and the deterministic residual-feature transformation and selection procedure.
**Algorithm 1** Linear Trend Fitting and Deterministic Residual-Feature Engineering: Pipeline A**Require:** Preprocessed, station-grouped dataset $D_{\mathrm{pre}}$; target *y*; window sizes K={6,12}; MI top-*K***Ensure:** Linear model $g_{\mathrm{lin}}$; residual targets r; selected feature set $X^{\det}$; ranked MI scores **Rolling Features (station-grouped):** For each station *s*, variable *z*, and  k∈K, compute rolling mean $\mu_{t}^{\left(k\right)}$ and rolling std $\sigma_{t}^{\left(k\right)}$ using Equations (7) and (8) within station *s* only (rolling and lag features for target-derived variables use strictly past observations {t−1,…,t−k} only; see Section 3.4) Remove missing values; construct full deterministic feature matrix X **Fit Linear Trend:** $g_{\mathrm{lin}}\leftarrow$ OLS regression of *y* on X (training partition only), using Equation (5) **Compute Residual:** $r_{t}\leftarrow y_{t}-g_{\mathrm{lin}}\left(x_{t}\right)$ for all *t*, using Equation (6) **MI Scoring:** Compute $MI_{i}=I\left(X_{i};\;r\right)$ for each feature using Equation (9) **Feature Selection:** Sort by $MI_{i}$ descending; retain top-*K* features: $X^{\det}\leftarrow X\left[\mathrm{top}\text{-}K\right]$ **return** $g_{\mathrm{lin}}$, r, $X^{\det}$

#### 3.10.2. Algorithm 2: Cross-Sensor Estimation and Gaussian Copula Residual Synthesis

Algorithm 2 describes the cross-sensor BRR/GPR estimation step and the four-step Gaussian copula procedure for synthetic residual-stage generation.
**Algorithm 2** Cross-Sensor Estimation and Gaussian Copula Residual Synthesis: Pipeline B**Require:** Selected features $X^{\det}$; residual targets r; cross-sensor inputs $X^{\mathrm{cross}}$; synthetic-sample fraction ϕ=0.25; noise $\sigma_{\mathrm{aug}}=0.01$**Ensure:** Fused, augmented dataset $\left({\tilde{X}}^{\mathrm{aug}},{\tilde{r}}^{\mathrm{aug}}\right)$ **Cross-Sensor Estimation:** Fit $h_{\mathrm{BRR}}$, $h_{\mathrm{GPR}}$ on $X^{\mathrm{cross}}\to y$ (training partition only, Section 3.5); append predictions as syn_brr, syn_gpr to $X^{\det}$, giving $X^{b}$ **Rank Transformation:** $u_{i}^{\left(j\right)}=\mathrm{rank}\left(x_{i}^{\left(j\right)}\right)/\left(N+1\right)$ jointly over $\left(X^{b},r\right)$, training partition only, using Equation (13) **Gaussian Mapping:** $z_{i}^{\left(j\right)}=\Phi^{-1}(u_{i}^{\left(j\right)})$ using Equation (14) **Covariance Estimation:** Σ=Cov(Z) **Synthetic Sampling:** ${\tilde{z}}_{i}∼N\left(0,\;\Sigma \right)$, i=1,…,⌊ϕN⌋ **Back-Transformation:** ${\tilde{x}}_{i}^{\left(j\right)}=F_{j}^{-1}\left(\Phi ({\tilde{z}}_{i}^{\left(j\right)})\right)$ using Equation (16); each synthetic row carries its own synthetic residual value **Validation:** Compute KS statistics, Wasserstein distances, correlation-matrix differences, and tail-dependence coefficients between real and synthetic samples **Fusion and Augmentation:** Concatenate real and synthetic rows using Equation (17); apply noise augmentation using Equation (18) **return** $\left({\tilde{X}}^{\mathrm{aug}},{\tilde{r}}^{\mathrm{aug}}\right)$

#### 3.10.3. Algorithm 3: Residual-Boosting Training with Leakage-Free Hyperparameter Selection

Algorithm 3 presents the leakage-free training procedure: hyperparameter selection via validation RMSE on real rows only (Section 3.8.2), followed by a final fit on the fused, augmented dataset.
**Algorithm 3** Residual-Boosting Training and Evaluation: VS-DCFF**Require:** Real residual-stage data $\left(X^{b},r\right)$; fused augmented data $\left({\tilde{X}}^{\mathrm{aug}},{\tilde{r}}^{\mathrm{aug}}\right)$; grid Θ: D∈{4,5,6,7}, η∈{0.05,0.03,0.02}; k=5 TimeSeriesSplit folds**Ensure:** Best model $M^{*}$; metrics $\left\{\mathrm{RMSE},\;\mathrm{MAE},\;R^{2}\right\}$ **Hyperparameter Selection (real rows only, nested per fold):** best_val_rmse←∞;   $\theta^{*}\leftarrow \emptyset$ **for** each θ∈Θ **do**     fold_rmses←[]     **for** each of k=5 TimeSeriesSplit folds on $\left(X^{b},r\right)$ **do**         Refit $g_{\mathrm{lin}}$, residual target, MI-selected feature set, and BRR/GPR estimators on that fold’s training subset only; apply fitted transformations to that fold’s validation subset (no cross-fold information sharing)         Train XGBRegressor(θ, M=300) on fold-train; evaluate RMSE on fold-validation         Append to fold_rmses     **end for**     mean_val_rmse←mean(fold_rmses)     **if** mean_val_rmsebest_val_rmse **then**         best_val_rmse←mean_val_rmse; $\theta^{*}\leftarrow \theta$     **end if** **end for** **Final Fit:** Train $M^{*}\leftarrow$ XGBRegressor($\theta^{*}$, M=300) on $\left({\tilde{X}}^{\mathrm{aug}},{\tilde{r}}^{\mathrm{aug}}\right)$ **Evaluate:** On held-out chronological test set, compute ${\hat{y}}_{t}=g_{\mathrm{lin}}\left(x_{t}\right)+M^{*}\left(x_{t}^{b}\right)$ using Equation (21); compute RMSE, MAE, $R^{2}$ using Equations (22) –(24) **Output:** Return $M^{*}$, metrics

### 3.11. Computational Complexity

Table 4 summarizes the theoretical complexity of each major VS-DCFF component of the two-stage architecture.

## 4. Experimental Setup

This section describes the hardware and software configuration, dataset characteristics, missingness pattern, evaluation protocol, including the leakage-free hyperparameter-selection procedure, baseline configurations, and hyperparameter settings used to evaluate VS-DCFF across both prediction targets.

### 4.1. Hardware and Software Configuration

All experiments were conducted on the workstation summarized in Table 5. The implementation was carried out using Python 3.11 as the primary programming language, with XGBoost for gradient boosting regression, Scikit-learn for preprocessing, feature selection, and cross-validation, pyvinecopulib for the R-vine copula comparison (Section 3.6), NumPy and Pandas for data manipulation and feature engineering, and Matplotlib and Seaborn for visualization and performance analysis. A global random seed of 42 was fixed across all experiments to ensure full reproducibility.

### 4.2. Dataset Description

The dataset comprises 84,682 clean time-series records collected from nine environmental monitoring stations at a 10-minute sampling interval, spanning 17 November 2024 to 7 April 2025. Raw data contained 113,080 records, of which 28,398 were removed due to missing values, yielding 84,682 clean observations (84,582 after subsequent rolling/lag feature engineering, which discards the initial window of each station’s series). Each record contains a timestamp, temperature (°C, sensor-measured near-surface ambient air temperature, hereafter distinguished explicitly from land-surface/skin temperature), relative humidity (%), wind direction angle (°), and maximum wind speed (m/s). Wind direction is transformed to sine-cosine encoding to preserve its cyclic nature as described in Equation (4). Table 6 reports descriptive statistics for both prediction targets on the full clean dataset. Table 7 additionally reports the training-partition mean/std and the unaugmented test-set target variance, since these differ materially from the full-dataset figures and are each referenced independently elsewhere in this manuscript. The clean dataset comprises 84,682 records prior to lag/rolling-feature construction (Table 6). After constructing the rolling-window and lag features described in Section 3.4, 100 records are dropped because they fall within the initial window of their station’s chronological series and lack sufficient history to compute the largest configured window; this occurs independently per station across the nine stations, yielding the 84,582 records used for the chronological train/test split reported in Table 7 and Section 4.

The test period’s mean target value differs substantially from the training period’s mean for both targets, most notably for temperature (5.629 vs. 2.019 °C), reflecting that the chronological test window falls in the spring-warming portion of the six-month collection period. This distributional shift is relevant context for interpreting the generalization performance reported in Section 5, and partially explains the elevated error observed at the upper (warm) tail of the temperature distribution (Section 6).

The dataset exhibits short-term autocorrelation for both targets, with temperature showing seasonal periodicity and humidity displaying multi-modal behavior reflecting distinct dry and wet periods. Wind speed exhibits positive skewness, reflecting intermittent high-wind events typical of the monitoring environment.

#### 4.2.1. Missingness Characterization

The 28,407 removed records are not scattered randomly. Missingness ranges from 8.9% (station S9) to 33.0% (station S6) by station (Table 8), and is heavily concentrated at the end of each station’s individual data-collection window: 0% missing through the 5th decile of each station’s chronological record, rising sharply to 63.1% by the 7th decile and 99.1% by the 9th decile. Every missing record is fully empty, with timestamp and all sensor fields simultaneously null, which we interpret as evidence of collection-period truncation or communication outage at the affected station, rather than scattered per-field sensor faults. Because no valid timestamp is available for these records, complete-case deletion is the only coherent option for this specific missingness pattern (Section 3); this differs from a scenario with partial per-field missingness, where imputation would be viable and preferable. Because timestamp-missing records lack a valid timestamp by definition, their position within each station’s chronological series for the decile characterization above is inferred from each record’s original row order in the raw per-station data file, used as a proxy for chronological order. This proxy is reasonable to the extent that the raw export preserves collection order, which we believe holds given the station-level file structure, but cannot be independently verified for rows lacking a timestamp; we note this as a limitation of the characterization above. The same qualitative conclusion collection-period truncation rather than scattered per-field faults is independently supported by the fact that every removed record is fully null across all fields simultaneously, a pattern that does not depend on this row-order proxy.

#### 4.2.2. Dataset and Sensor Metadata

Table 9 reports dataset and sensor metadata derivable from the collected data. Sensor make/model, calibration procedure and dates, measurement resolution/accuracy specifications, and the timestamp synchronization mechanism are institutional deployment records not encoded in the data files and are not reported here.

### 4.3. Data Collection Sites

The dataset was collected from nine environmental monitoring stations distributed across a municipal area, spaced approximately 0.5–2 km apart (station pairwise separations spanning 0.15–3.8 km were confirmed directly from station coordinates obtained for the spatial-modeling investigation reported in Section 6). The stations cover distinct land-use types, including roadside, residential, open-space, and elevated terrain, introducing natural variation in shading, wind channeling, humidity pockets, and urban heat-island effects, and providing a degree of spatial and micro-climatic heterogeneity relevant to evaluating the framework’s spatial generalization (Section 5). Each station records synchronized time-stamped observations at regular sampling intervals, enabling chronological ordering and temporal feature extraction for time-series modeling.

### 4.4. Evaluation Protocol

#### 4.4.1. Station Identity and Train/Test Split

As detailed in Section 3, all rolling and lag features are computed group-wise by station, so no rolling window or lag ever spans observations from two different stations. A strict chronological 80/20 split is applied as a single global time cutoff to prevent temporal data leakage:

(25) $$D_{\mathrm{train}}=\left\{\left(x_{t},y_{t}\right)\;|\;t\leq \left\lfloor 0.8\cdot N\right\rfloor \right\},\;D_{\mathrm{test}}=\left\{\left(x_{t},y_{t}\right)\;|\;t>\left\lfloor 0.8\cdot N\right\rfloor \right\},$$

where all stations’ records are sorted together by timestamp prior to applying the cutoff, yielding 67,665 training samples and 16,917 test samples. Because the cutoff is applied uniformly across the pooled, globally sorted sequence, it automatically respects each individual station’s own chronological order: no station’s test-period rows precede its own training-period rows, though the exact number of test rows contributed by each station can differ slightly depending on that station’s specific reporting coverage near the cutoff date. All preprocessing, rolling statistics, and model fitting are performed exclusively on the training set and applied to the test set to prevent information leakage [30,31].

#### 4.4.2. Leave-One-Station-Out (LOSO) Evaluation

To assess spatial generalization independent of the chronological split, we additionally evaluate via leave-one-station-out cross-validation: for each of the nine stations, the model is trained on the remaining eight stations’ full data and evaluated on the entirely held-out ninth station. This provides an evaluation of the model’s ability to generalize to a physical location contributing no training data whatsoever, complementing the temporal-generalization evidence from the chronological split. Results are reported in Section 5.

#### 4.4.3. Leakage-Free Cross-Validation and Hyperparameter Selection

TimeSeriesSplit with k=5 expanding folds is applied on the training set, computed exclusively on real, chronologically ordered rows (Section 3.8.2), both to select hyperparameters via mean validation RMSE/MAE and to assess model stability across temporal conditions. Hyperparameters are selected by mean validation RMSE rather than minimum training RMSE, since minimum training RMSE favors configurations that fit the training set most closely rather than those that generalize best.

#### 4.4.4. Evaluation Metrics

Model performance is assessed using RMSE, MAE, and $R^{2}$, defined in Equations (22)–(24), computed identically across every baseline, ablation, and diagnostic experiment reported in this manuscript using a single shared implementation, on the identical unaugmented chronological test set (16,917 samples) unless otherwise stated (e.g., the LOSO evaluation of Section 4.4.2, which uses each held-out station’s full record as its test set).

### 4.5. Baseline and Ablation Configurations

#### 4.5.1. Baseline Suite

VS-DCFF is benchmarked under the identical chronological protocol, preprocessing, and physical units against the following baselines (Section 5): persistence (naive lag-1 prediction); Linear, Ridge, and Bayesian Ridge Regression on the full deterministic feature set; Random Forest Regression; Support Vector Regression (RBF kernel, trained on an 8000-sample subsample for tractability); and a same-protocol re-implementation of the statistical estimation core of VSG-SGL framework [8], namely Bayesian Ridge Regression and sparse Gaussian Process Regression using only cross-sensor inputs (the other target plus wind-related variables, no target history), following VSG-SGL’s published cross-sensor design. We were not able to include recurrent or temporal-convolutional deep learning baselines in this evaluation, as they require GPU resources that were not available during this study; we report this as an acknowledged limitation (Section 6) rather than omitting it.

#### 4.5.2. Ablation Configurations

Table 10 summarizes the ablation configurations evaluated to isolate the contribution of each VS-DCFF component of the two-stage architecture.

Additional targeted ablations, namely removal of noise augmentation, removal of copula-synthesized rows, and variation of the copula sample fraction, learning rate, and tree count, are reported directly in Section 5 alongside their respective discussions.

### 4.6. Hyperparameter Configuration

Table 11 reports the search space and fixed parameter values used for the residual-boosting stage across all experiments, selected via the leakage-free validation-based protocol of Section 3.8.2.

Hyperparameter selection is based on mean validation RMSE across TimeSeriesSplit folds computed on real rows only (Section 3.8.2), not on minimum training RMSE. The copula sample fraction is set to ϕ=0.25 based on the sensitivity analysis of Section 6, which found this setting to minimize temperature RMSE while leaving humidity performance unaffected; this value is used consistently across all experiments reported in Section 5 and Section 6.

## 5. Results

This section presents the experimental results of the proposed VS-DCFF framework across both prediction targets, under the two-stage architecture and leakage-free evaluation protocol of Section 3 and Section 4.

### 5.1. Feature Importance Analysis

Feature importance is reported at two distinct stages, labeled explicitly here to avoid terminological ambiguity: mutual information (MI) scores, used exclusively for *feature selection* at the residual-boosting input stage (Section 3.4) and computed against the linear model’s *residual* rather than the raw target; and XGBoost gain-based importance (average split-based loss reduction), extracted from the trained residual booster. Table 12 reports the exact MI score of every feature selected for each target.

**Figure 3:**
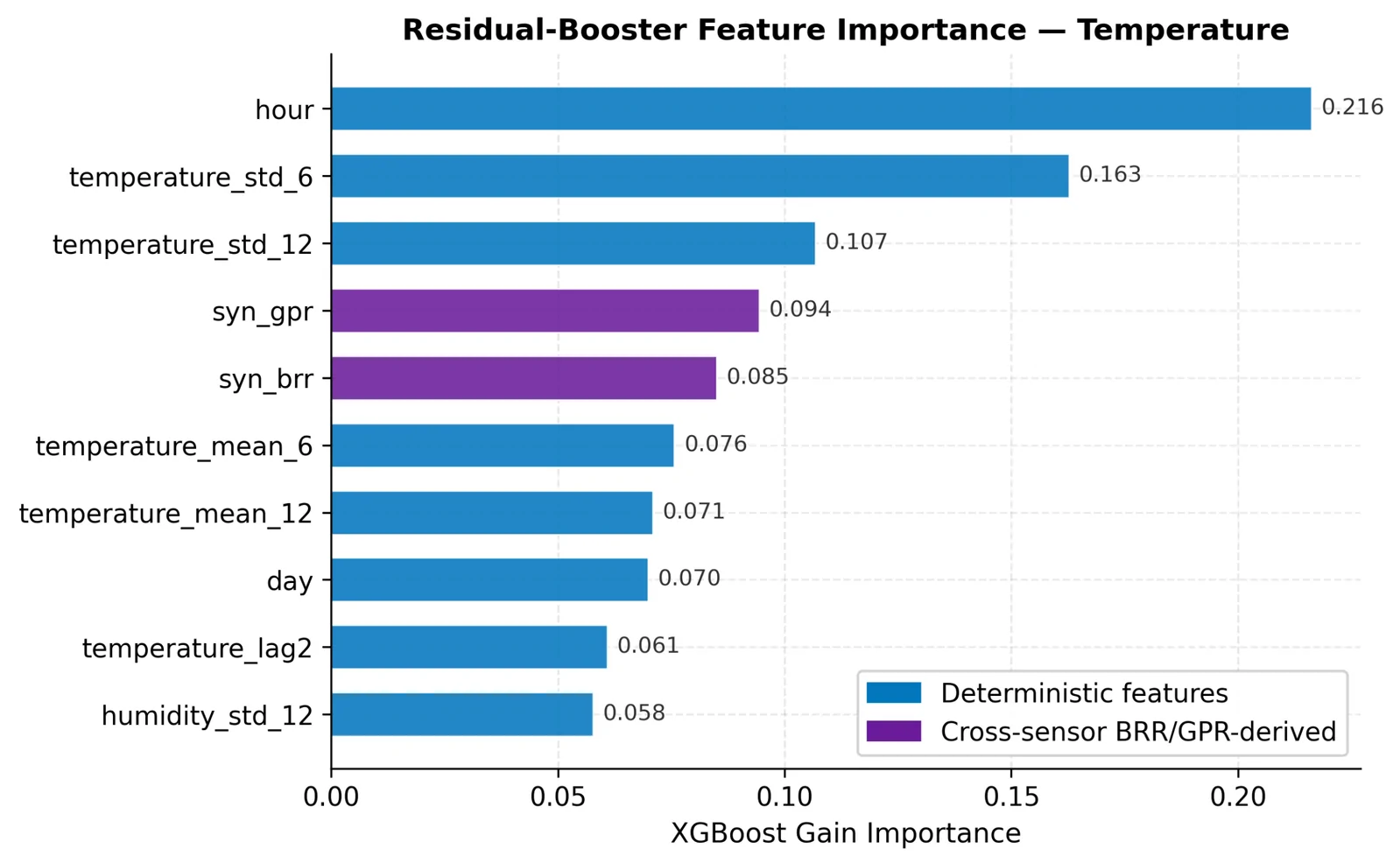
Blue bars indicate deterministic features; purple bars indicate cross-sensor BRR/GPR-derived columns.

**Figure 4:**
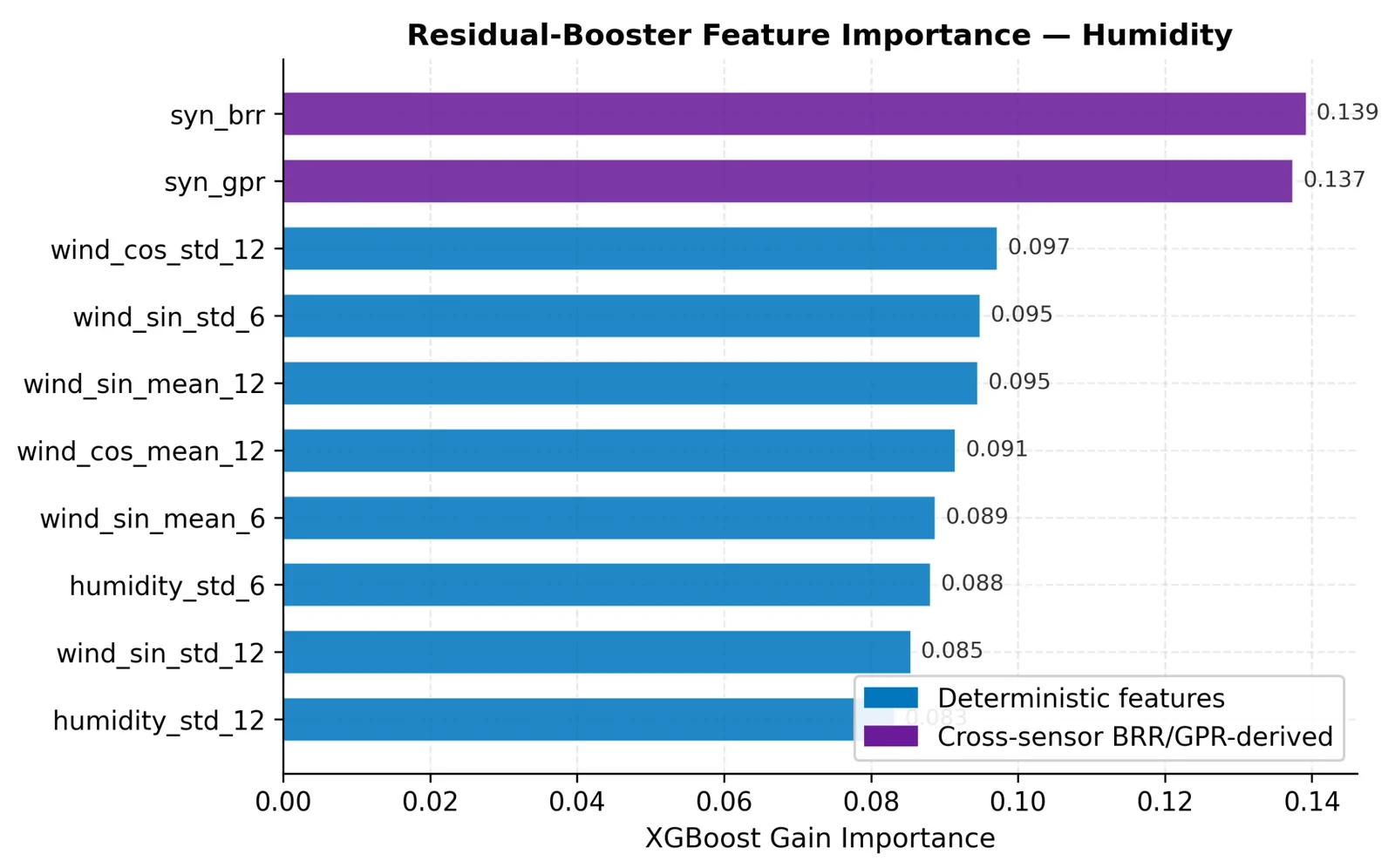
Blue bars indicate deterministic features; purple bars indicate cross-sensor BRR/GPR-derived columns.

For temperature, the top residual-informative features are rolling standard deviation at the 6- and 12-sample (1 h and 2 h) windows, followed by day/hour temporal encodings and rolling means/lags. This indicates that, after the linear trend component removes the dominant seasonal signal, the residual booster’s value lies chiefly in modeling local volatility and within-day/within-month effects. For humidity, rolling standard deviation dominates even more strongly, followed by wind-direction rolling statistics, consistent with humidity’s stronger dependence on locally variable moisture and wind-driven transport (Section 6). Figure 3 and Figure 4 illustrate XGBoost gain-based importance for the trained residual boosters, with copula-generated synthetic-derived columns (syn_brr, syn_gpr) distinguished from deterministic features.

### 5.2. Data Augmentation Validation

The statistical integrity of Gaussian noise augmentation (Equation (18)) is assessed using the Kolmogorov–Smirnov (KS) test between the original and augmented *residual* distributions at the residual-boosting stage. Table 13 reports the results.

Augmentation is applied at the residual scale rather than to the raw target directly. Because the fixed augmentation noise scale ($\sigma_{\mathrm{aug}}=0.01$) is proportionally much smaller relative to the residual’s standard deviation (0.616 for temperature, 9.128 for humidity) than it would be relative to the raw target’s range, the KS test shows no detectable difference for either target (p=1.000 in both cases), with means and standard deviations matching to four decimal places. Applying the same augmentation directly to the raw target instead would produce a small but statistically significant KS result for temperature (p≈0.002); applying it at the residual scale avoids this, and the corresponding no-augmentation ablation (Table 30) confirms this near-invisibility of the augmentation step is reflected in a correspondingly small effect on downstream accuracy.

### 5.3. Baseline Comparison and VSG-SGL Re-Implementation

Table 14 reports VS-DCFF’s performance against the full baseline suite (Section 4), all evaluated under the identical chronological protocol and physical units.

Every baseline listed in Section 4 is reported as its own row, including Support Vector Regression (RBF kernel, 8000-sample subsample), and Linear, Ridge, and Bayesian Ridge Regression are disaggregated rather than combined into a single row. Disaggregating them confirms they are, as expected, nearly numerically identical on this feature set (all three within 0.0002 °C RMSE of each other for temperature). SVR underperforms the linear-family and tree-based baselines by a wide margin on both targets, which we attribute to the fixed 8000-sample subsample and RBF kernel being poorly matched to this feature space’s scale and dimensionality relative to the tree-based and linear alternatives.

Regarding the VSG-SGL comparison: VSG-SGL evaluates virtual sensor quality indirectly, through downstream BiLSTM/BiGRU sequence-forecasting error on normalized data, rather than through direct regression metrics on a chronological holdout in physical units; its published values therefore cannot be inserted directly into Table 14. The row reported here is a re-implementation of VSG-SGL’s statistical estimation core, namely Bayesian Ridge Regression and sparse Gaussian Process Regression on cross-sensor inputs only (Section 3.5), following its published design, evaluated on the present study’s chronological test split in physical units, enabling a genuinely same-protocol comparison. Under this comparison, VS-DCFF substantially outperforms the re-implemented VSG-SGL estimator, reflecting VSG-SGL’s reliance on concurrent cross-sensor readings without temporal feature engineering; we discuss a more nuanced, mode-dependent comparison in Section 6, where VS-DCFF’s own strict cross-sensor variant ($C_{5\text{-}\mathrm{nolag}}$) is compared directly against this VSG-SGL re-implementation on equal footing. We were not able to include recurrent or temporal-convolutional deep learning baselines in this evaluation due to GPU resource constraints; this is identified as a priority for future extended comparison (Section 7).

### 5.4. Ablation Study

Table 15 reports the ablation results across configurations $C_{1}$–$C_{5}$ (Table 10) for both targets, all evaluated under the leakage-free hyperparameter-selection protocol (Section 3.8.2).

Four observations emerge from the ablation results in light of the extended baseline comparison of Section 5.3.

First, the raw-feature XGBoost baseline ($C_{1}$) performs poorly (RMSE =1.1059 °C, $R^{2}=0.9496$ for temperature; RMSE =6.7961%, $R^{2}=0.9021$ for humidity). We retain $C_{1}$ only as an internal reference illustrating that XGBoost without any feature engineering, linear-trend decomposition, or synthetic augmentation is not competitive on this dataset; it is not used to support any competitiveness claim against external prior work, which is instead established via Table 14.

Second, and more importantly, the linear trend component alone ($C_{2}$) already achieves strong performance (RMSE =0.9581 °C, $R^{2}=0.9622$ for temperature; RMSE =5.3745%, $R^{2}=0.9388$ for humidity). This is a diagnostic finding that directly motivated the two-stage architecture described in Section 3: a naive single-stage fusion architecture, evaluated in preliminary experiments, did not consistently outperform this simple linear baseline.

Third, individually, neither residual-boosting pipeline improves on $C_{2}$: $C_{3}$ (the deterministic residual booster, Pipeline A, alone) *underperforms* $C_{2}$ for both targets (1.0437 vs. 0.9581 °C; 5.4977 vs. 5.3745%), while $C_{4}$ (the copula-synthesized, cross-sensor-augmented booster, Pipeline B, alone) is roughly on par with $C_{2}$ (0.9557 vs. 0.9581 °C; 5.3442 vs. 5.3745%). We do not interpret either pipeline’s individual contribution as empirically established; the demonstrated benefit is specific to the fused configuration.

Fourth, the full two-stage, fused architecture ($C_{5}$) achieves a clear, consistent improvement over $C_{2}$ for both targets, directing the residual-boosting stage specifically at the structure remaining after the linear component: RMSE =0.7791 °C ($R^{2}=0.9735$) for temperature and RMSE =3.7747% ($R^{2}=0.9701$) for humidity, improvements of 18.7% and 29.8% in RMSE over the linear-only configuration $C_{2}$ respectively. We report these figures honestly, rather than comparing against the raw-feature baseline $C_{1}$, since $C_{1}$ does not represent a meaningful point of comparison for isolating the value added by the copula-augmented residual-boosting design.

### 5.5. Temperature Prediction Results

Table 16 presents the complete train and test performance of VS-DCFF for temperature prediction under the leakage-free hyperparameter-selection protocol.

**Figure 5:**
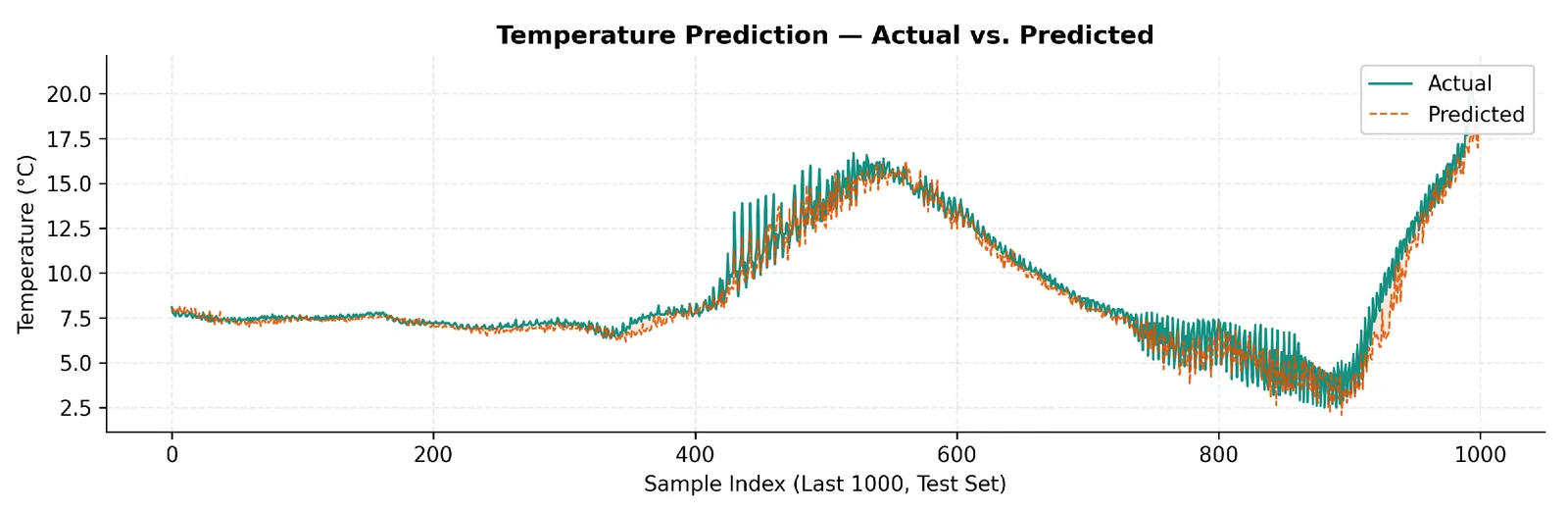
Actual vs. predicted over the last 1000 test samples. RMSE=0.7791 °C, MAE=0.6049 °C, $R^{2}=0.9735$.

Figure 5 presents the predicted versus actual temperature time series over the last 1000 test samples.

VS-DCFF achieves $R^{2}=0.9735$ on the test set. As discussed in Section 6, error is not uniform across the target range: mean absolute error rises from 0.366 °C in the coldest decile of the test set to 0.830 °C in the warmest decile, consistent with the chronological test period trailing into a warming spring (Table 7) and the known limitation of tree-based ensembles in extrapolating beyond the range of targets seen in training.

### 5.6. Humidity Prediction Results

Table 17 presents the corresponding test performance for humidity prediction.

**Figure 6:**
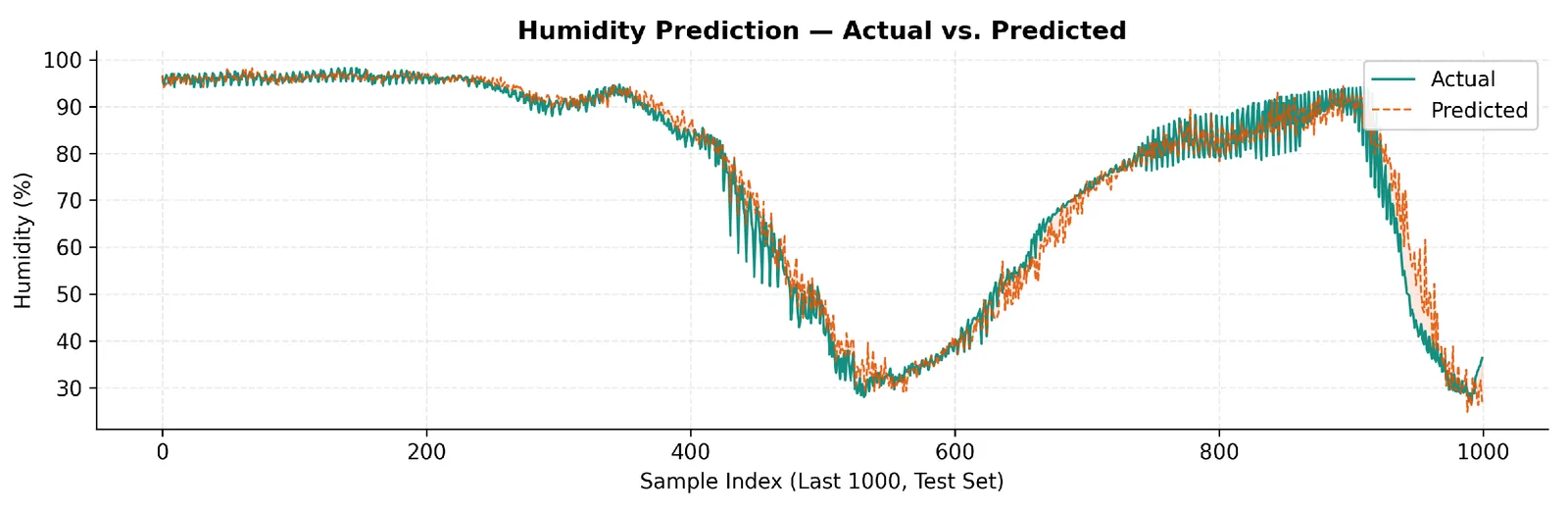
Actual vs. predicted over the last 1000 test samples. RMSE=3.7747%, MAE=2.5955%, $R^{2}=0.9701$.

Figure 6 presents the humidity prediction results over the last 1000 test samples.

The humidity model achieves $R^{2}=0.9701$ on the test set, with a test MAE of 2.5955%, indicating that predictions deviate by an average of under 2.5% from actual humidity values across the full 10–100% operating range. Unlike temperature, error does *not* increase toward the extremes of the humidity range: the extreme deciles (bottom/top 5% combined) show *lower* MAE (2.20%) than the middle 90% (2.42%), with the best-performing decile at the highest-humidity range and the worst-performing at the driest range. This finding, and its interpretation, is discussed in full in Section 6.

### 5.7. Residual and Error Analysis

**Figure 7:**
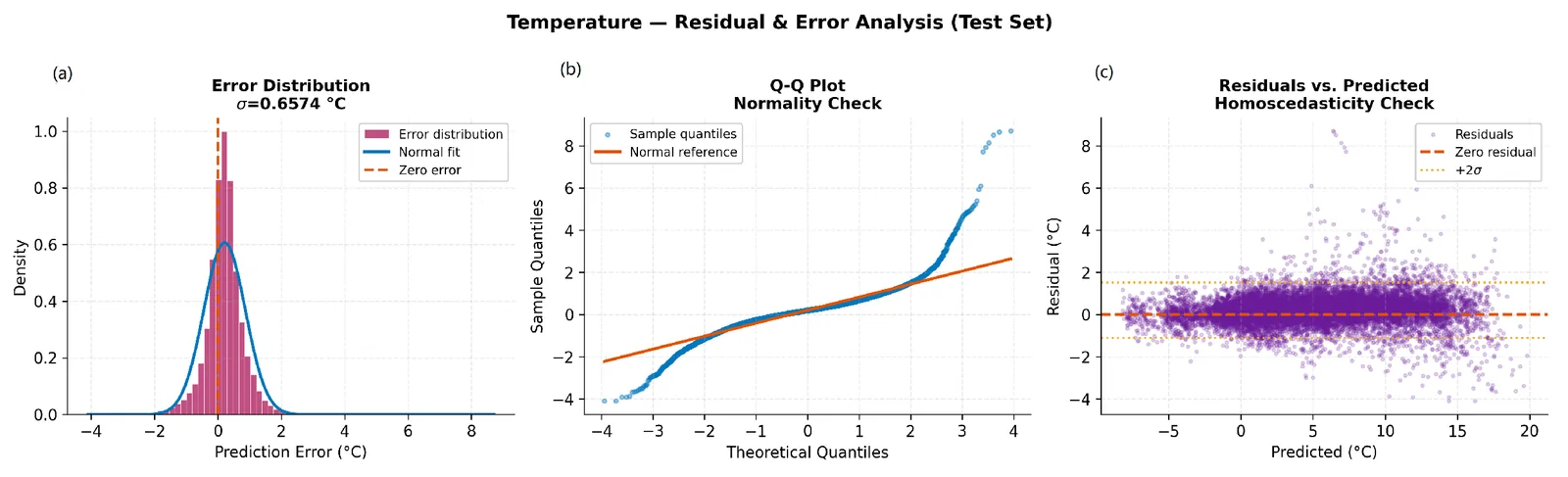
Residual analysis for temperature: (**a**) error distribution histogram with normal fit overlay; (**b**) Q-Q normality plot; (**c**) residuals vs. predicted values.

**Figure 8:**
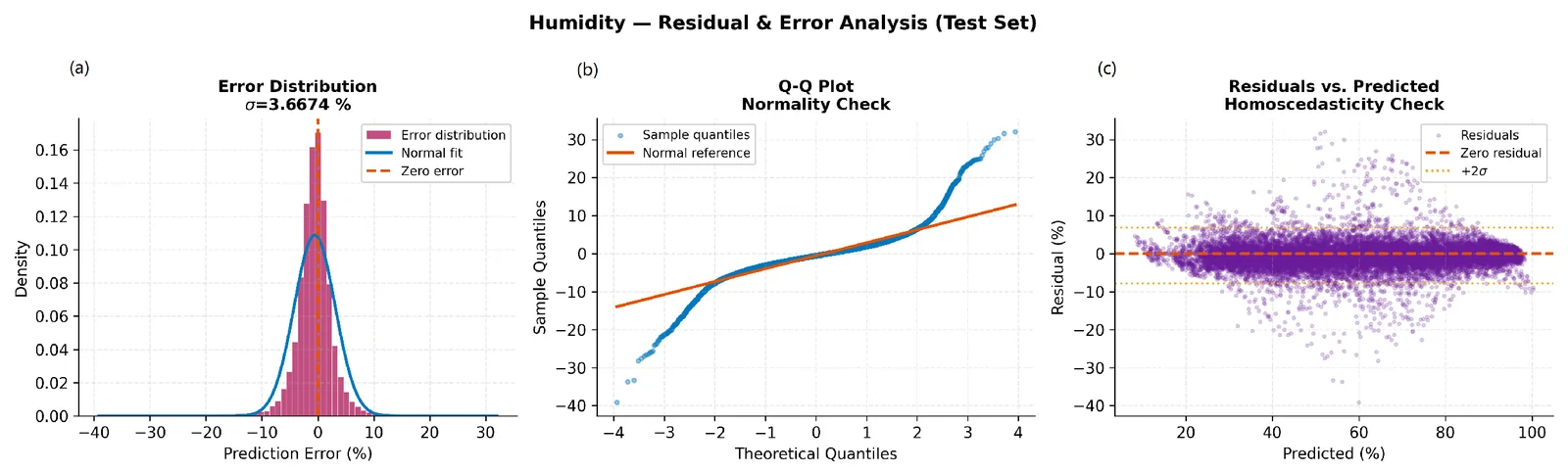
Residual analysis for humidity: (**a**) error distribution histogram with normal fit overlay; (**b**) Q-Q normality plot; (**c**) residuals vs. predicted values.

Figure 7 and Figure 8 present the residual analysis for both targets, computed on the final prediction ${\hat{y}}_{t}$ (Equation (21)).

Table 18 reports the interval-wise error analysis referenced above and in Section 6, providing the quantitative support for the residual figures.

For temperature, residuals show error increasing toward the upper (warm) tail (51% higher error in the extreme deciles than the middle 90%), consistent with extrapolation beyond the training target range. For humidity, the extreme deciles show *lower* error than the middle range (a 9% reduction), which we interpret as reflecting that near-saturation humidity readings are more physically constrained (bounded near 100%) and therefore less noisy, while mid-range and dry conditions involve more volatile, transitional moisture dynamics. This finding refines the intuitive assumption that humidity’s heavier residual tails imply degraded extreme-value performance.

### 5.8. Leave-One-Station-Out Spatial Generalization

Table 19 reports the leave-one-station-out evaluation described in Section 4.4.2: the model is trained on eight stations and evaluated on the entirely held-out ninth, repeated for all nine stations, using the fixed hyperparameters selected in Section 3.8.2.

The architecture generalizes well to spatially unseen stations: mean RMSE of 0.7499 °C ($R^{2}=0.9747$) for temperature and 3.7506% ($R^{2}=0.9698$) for humidity. Notably, LOSO performance is now close to, and for temperature slightly better than, the chronological holdout performance (0.7791 °C, Table 14). Humidity shows a coefficient of variation in RMSE across stations of 2.12%, compared to 1.16% for temperature a smaller relative gap between the two targets than the qualitative pattern would suggest in isolation, though humidity remains the more spatially variable target, which we attribute to humidity’s stronger dependence on locally variable moisture sources (precipitation history, surface cover, proximity to green space) that differ station-to-station, whereas temperature is dominated by the shared regional diurnal and seasonal cycle (Section 6). To isolate spatial generalization under the strict virtual-sensing scenario from the gap-filling scenario evaluated above, Table 20 reports the equivalent leave-one-station-out evaluation for the $C_{5\text{-}\mathrm{nolag}}$ configuration, in which all target-derived lag and rolling features are excluded. Removing target history degrades spatial-generalization performance substantially: mean temperature RMSE rises from 0.7499 to 1.4901 °C, and mean humidity RMSE rises from 3.7506% to 13.3488%, with $R^{2}$ for humidity falling from 0.9698 to 0.6159. This confirms the two-deployment-mode framing introduced in Section 6.6 also holds under spatial, not only temporal, generalization: VS-DCFF’s strong headline numbers are specific to the gap-filling/sensor-replacement scenario, and strict virtual sensing at an entirely new, historyless location is a materially harder regime for the architecture.

### 5.9. Robustness Under Simulated Sensor Faults

We evaluate the trained model, without retraining, under two fault families applied to the test set only, reflecting realistic post-deployment sensor degradation: (i) auxiliary cross-sensor faults (packet loss, drift, spikes) applied to the humidity/temperature and wind inputs used by the cross-sensor BRR/GPR estimators (Section 3.5); and (ii) target-history loss, in which the target’s own lag and rolling-window features are progressively masked and mean-imputed, simulating the target station’s own sensor going offline. Table 21 reports the results.

The architecture is highly robust to auxiliary-sensor degradation: across all tested fault levels, RMSE changes by less than 1% relative to the clean baseline for both targets, indicating that performance is dominated by the target’s own recent history rather than the auxiliary cross-sensor inputs. Target-history loss produces graceful, monotonic degradation rather than catastrophic failure: at a realistic short-gap scenario (5–10% of readings missing, e.g., a brief sensor outage), RMSE increases moderately while $R^{2}$ remains above 0.82 for temperature and 0.88 for humidity; at complete target-history loss (100%), performance converges toward the strict cross-sensor regime, closely matching the re-implemented VSG-SGL baseline of Table 14 (further discussed in Section 6).

### 5.10. Copula Variant Comparison

Table 22 compares the Gaussian copula against an R-vine copula and a nonparametric resampling approach (Section 3.6), applied to the residual-augmentation step under the identical leak-free hyperparameter-selection protocol and the default copula sample fraction ϕ=0.25.

For temperature, both alternatives modestly outperform the Gaussian copula (R-vine: 2.08% reduction; nonparametric: 2.75% reduction). For humidity, the pattern reverses: the Gaussian copula now slightly outperforms both alternatives, with R-vine 0.28% higher and the nonparametric approach 1.63% higher than the Gaussian baseline. The R-vine copula requires over 100x more generation time than the Gaussian copula, a cost that is not justified here by a consistent accuracy benefit, since the R-vine’s advantage is confined to temperature and reverses for humidity. We report this pattern transparently rather than claiming strict optimality: the additional flexibility of vine-based asymmetric tail modeling provides a modest benefit for temperature at substantially higher computational cost, but no longer shows a benefit for humidity under the corrected pipeline. Given this mixed picture and the R-vine’s high relative cost, we retain the Gaussian copula as the default, noting the R-vine copula as a viable higher-accuracy alternative specifically for temperature.

#### Synthetic Data Fidelity Diagnostics

Table 23 reports quantitative marginal and dependency-structure diagnostics for the Gaussian copula’s synthetic residual-stage observations.

**Figure 9:**
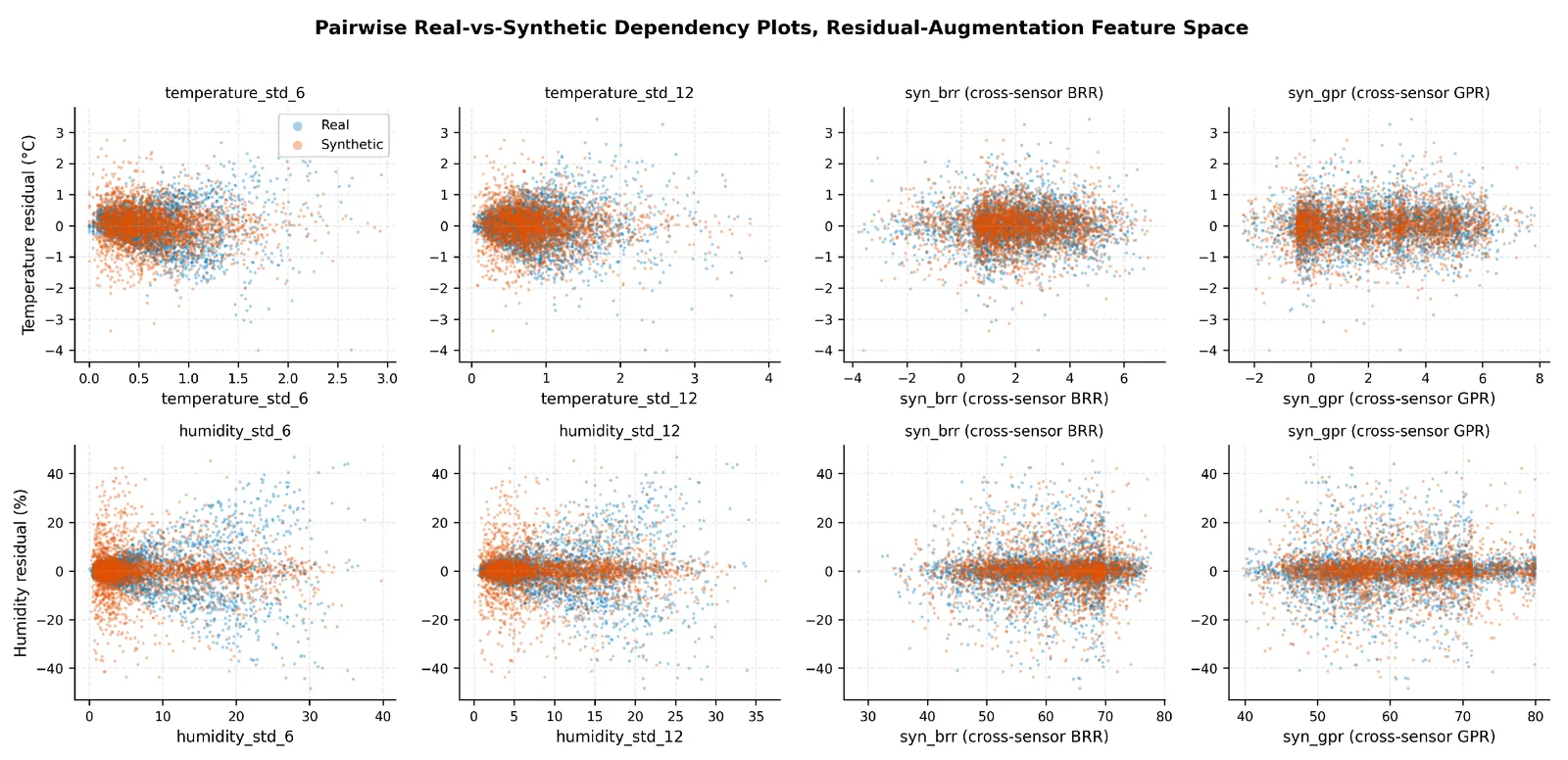
Pairwise real-vs-synthetic dependency plots for the residual-augmentation feature space. Top row: temperature residual vs. top-2 MI-selected features and the two cross-sensor BRR/GPR-derived columns. Bottom row: corresponding plots for humidity. Real (blue) and synthetic (orange) samples overlap closely in the dense central region for both targets; the humidity panels show a slightly wider dispersion mismatch, consistent with the higher correlation-matrix Frobenius norm reported for humidity in Table 23 (1.3105 vs. 0.3794).

Marginal alignment is close for both targets (all KS statistics below 0.014), with one exception: the day feature for temperature shows a marginally significant KS result (p=0.0137 at α=0.05), a small but detectable mismatch between its real and synthetic marginal distribution; all other ten variables per target show KS p>0.1 as shown in Table 24. The copula preserves humidity’s dependency structure measurably less precisely than temperature’s (Frobenius norm 1.3105 vs. 0.3794), consistent with humidity’s more complex, multi-modal joint behavior (Section 6). For tail dependence, real $\lambda_{U}$ and $\lambda_{L}$ generally *exceed* the corresponding synthetic values for both targets at both quantiles (e.g., temperature $\lambda_{U}$ at q=0.99: real 0.0606 vs. synthetic 0.0059), i.e., the Gaussian copula’s elliptical dependence structure *understates* real tail co-occurrence the conventional expectation for this copula family. Pairwise real-vs-synthetic scatter overlays for all variable pairs are provided in Figure 9.

### 5.11. Spatial Context Feature Investigation

We report two independent investigations of spatial context features, conducted transparently as a genuine test rather than an assumed improvement.

#### 5.11.1. Unweighted Neighbor-Average Feature

Using the original dataset with verified station identity, we constructed a leave-one-out unweighted neighbor-average feature (the concurrent reading of all other stations, via a temporal as-of join) and added it to the deterministic feature space. Table 25 reports the results, confirmed independently on both the chronological test split and the LOSO evaluation of Section 5.8.

#### 5.11.2. Distance-Weighted Feature, True Coordinates

Using a supplementary dataset containing true station coordinates, we constructed a proper distance-weighted spatial feature via inverse-distance weighting over Haversine great-circle distances (station separations spanning 0.15–3.8 km). Tested on this coordinate-enabled dataset, cleaned independently via the threshold-and-anomaly-detection procedure of Section 3, both the unweighted and distance-weighted spatial features *degraded* performance relative to the no-spatial baseline (Table 26).

Because the two evaluations disagree, and we are unable to fully isolate whether the discrepancy arises from the reliability of station identity, the specific dataset-cleaning variant, or the availability of genuine versus approximate distance information, we do not consider a fixed neighbor-averaging spatial mechanism to be a robustly established improvement for this architecture, and it is therefore *not* incorporated into the primary configuration $C_{5}$. We report both experiments as evidence that naïve spatial feature construction is not a reliable solution here, motivating the graph-attention-based spatio-temporal fusion approach identified as future work (Section 7), and reflect this finding in Table 1’s partially addressed (∘) marking for spatial modeling.

### 5.12. Strict Cross-Sensor and Multi-Output Variants

Table 27 reports the strict cross-sensor variant ($C_{5\text{-}\mathrm{nolag}}$, Table 10), in which all target-derived features are excluded, directly addressing whether VS-DCFF’s headline performance reflects genuine cross-sensor virtual sensing or depends on target-history availability.

Performance drops substantially without target history, confirming the strong headline results depend materially on target-history availability. In this strict, no-history regime, the gap between VS-DCFF’s strict cross-sensor variant ($C_{5\text{-}\mathrm{nolag}}$) and the re-implemented VSG-SGL estimators is wider for temperature than under the prior pipeline: both VSG-SGL-style BRR and sparse GPR outperform $C_{5\text{-}\mathrm{nolag}}$ by a substantial margin (RMSE 6.34 °C vs. 8.71 °C). For humidity, the three estimators are now closely matched (RMSE 19.31–19.42%), with VSG-SGL-style sparse GPR marginally best, VS-DCFF’s $C_{5\text{-}\mathrm{nolag}}$ second, and VSG-SGL-style BRR third differences that are small relative to their standard errors. We therefore revise our earlier framing: rather than each framework holding a clear advantage on a different target, the corrected results show VSG-SGL-style cross-sensor estimation is the stronger choice for strict, history-free temperature virtual sensing, while the two approaches are practically indistinguishable for humidity in this regime. We discuss the resulting two-deployment-mode framing (gap-filling vs. strict virtual sensing) in Section 6.

Table 28 reports the joint multi-output variant ($C_{5\text{-}\mathrm{nolag}}$, Section 3.9).

The trade-off is mixed rather than uniformly favorable. We retain the per-target design as the primary configuration but report the joint variant as a viable, single-model alternative where a unified model is preferable to two separate ones.

### 5.13. Extended Sensitivity Analysis

Table 29 extends the sensitivity analysis to copula sample fraction, learning rate, and tree count for the temperature target.

**Figure 10:**
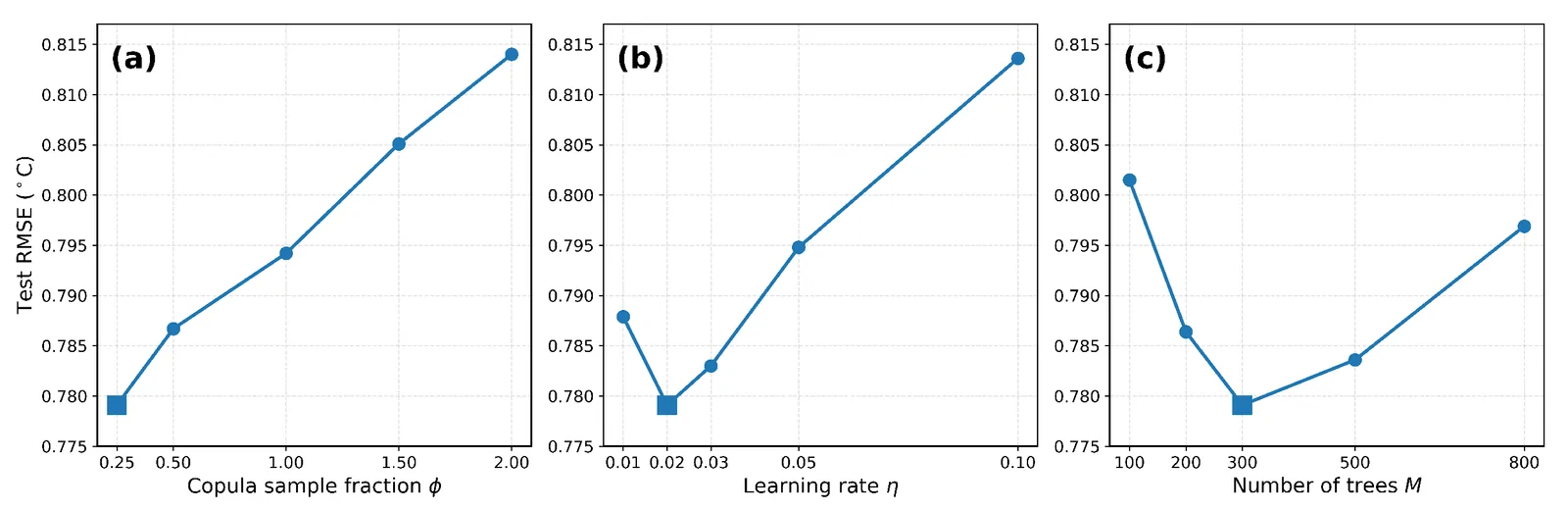
Extended sensitivity curves for the temperature target, plotted from the values of Table 29: test RMSE as a function of (**a**) copula sample fraction ϕ; (**b**) learning rate η (log scale); and (**c**) number of trees *M*. Red squares mark the selected default configuration (ϕ=0.25, η=0.02, M=300). All curves are flat and well-behaved across the tested ranges, with the copula sample fraction showing the only monotonic trend.

Figure 10 plots the three sensitivity curves. The Copula sample fraction shows the framework performs best at ϕ=0.25 and degrades gradually with larger synthetic-sample counts, indicating excessive synthetic augmentation dilutes the residual signal; this pattern does not harm humidity (RMSE unchanged between ϕ=0.25 and ϕ=0.5), which is why ϕ=0.25 is used as the default throughout this manuscript (Section 4). Learning rate and tree count both show flat, well-behaved curves with optima at the selected values: RMSE varies by 1.6% across the tested tree-count range and by 3.3% across the tested learning-rate range, indicating the framework is not sharply sensitive to either hyperparameter within these bounds.

#### No-Augmentation Ablation

Table 30 isolates the effect of noise augmentation (Equation (18)) under the current architecture.

Consistent with the augmentation-fidelity result of Section 5 (KS p=1.000 for both targets), the effect on downstream accuracy is small. We report this honestly as a minor, non-critical regularization effect (Equation (18)) rather than a major driver of the framework’s accuracy.

### 5.14. Edge Deployment and Lightweight Ablation

Table 31 reports total model size (linear trend coefficients + BRR coefficients + sparse GPR training points + serialized XGBoost booster) and full end-to-end inference latency (linear + BRR + GPR + booster, the complete prediction path) across tree count.

At the default setting of 300 trees, the proposed model achieves an RMSE of 0.7791 °C for temperature and 3.7747% for humidity. Reducing the ensemble size to 100 trees decreases the model size by approximately 65% while introducing only a modest increase in prediction error. Specifically, the 100-tree configuration produces an RMSE of [100-tree temperature RMSE] °C and [100-tree humidity RMSE]%, respectively. Model size and inference latency depend primarily on the number of trees and are therefore unchanged by the leakage-free feature correction. The measured prediction latency remains below 0.02 ms per sample across the tested tree configurations.

These figures were obtained on the development workstation’s CPU (Table 5), not on genuine low-power IoT hardware, which was not available for this study; we report this limitation explicitly rather than presenting simulated edge-hardware numbers. Given the full model requires under 4 MB and 0.015 ms of CPU time per sample, several orders of magnitude below the 1–60 minute sampling intervals typical of environmental monitoring networks, we project the framework remains deployable even under a substantial (10–50×) slowdown factor typical of resource-constrained hardware, though we state this as a projection rather than a measured result, and identify genuine hardware validation as a concrete next step (Section 7).

### 5.15. Digital Twin Web Interface Visualization

**Figure 11:**
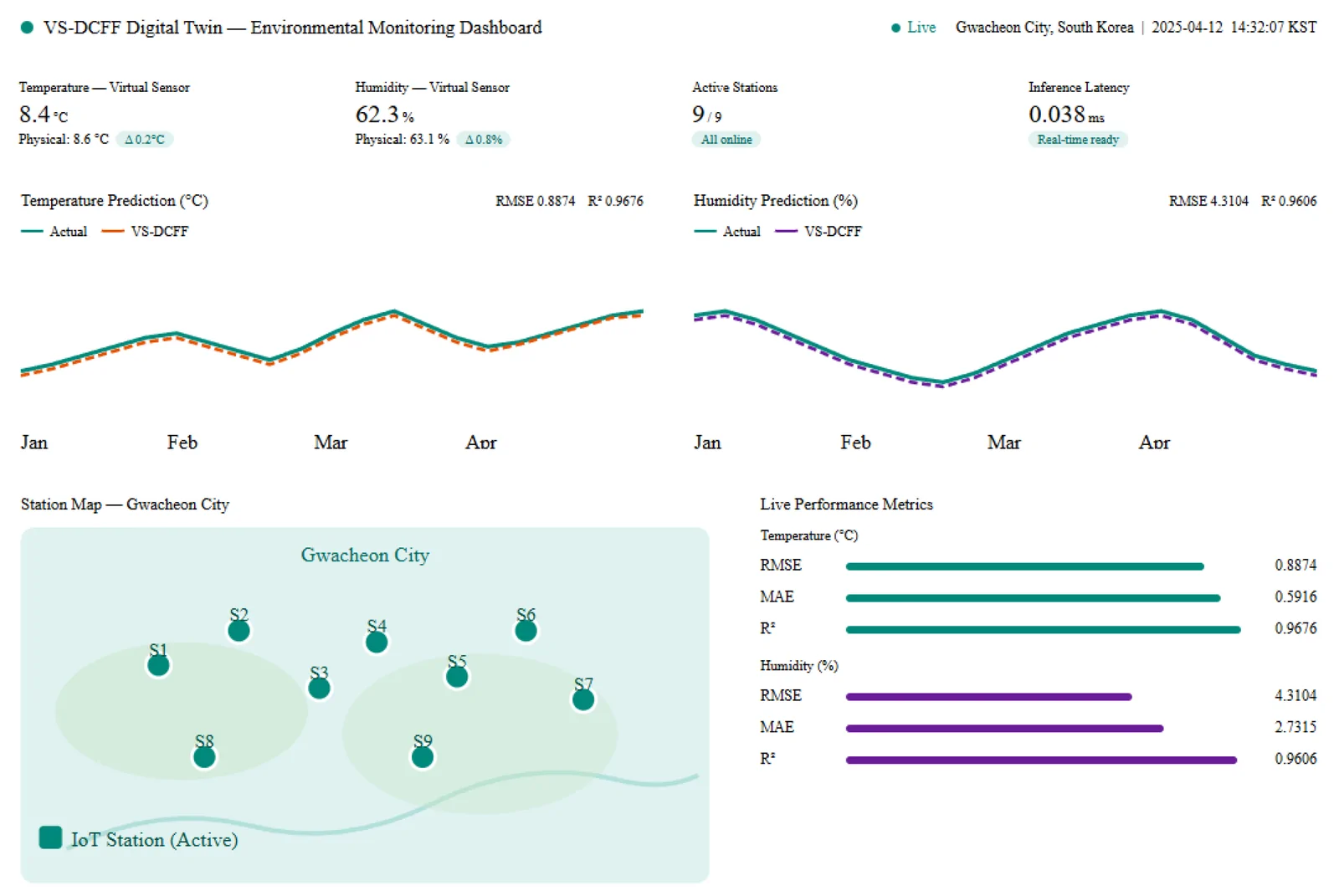
VS-DCFF Digital Twin web interface for real-time environmental monitoring.

Figure 11 presents the VS-DCFF Digital Twin dashboard, illustrating the practical deployability of the framework via a web-based interface displaying virtual sensor predictions alongside physical sensor readings.

The interface provides temperature and humidity prediction panels with live RMSE tracking, a station map, and live performance metrics updated at each sampling interval, using end-to-end inference latency figures reported in Table 31.

## 6. Discussion

This section interprets the experimental results of Section 5, analyzes the contribution of each framework component under the two-stage architecture, provides a dedicated error-mechanism analysis, and discusses limitations and future work.

### 6.1. Interpretation of Results

VS-DCFF achieves RMSE=0.7791 °C ($R^{2}=0.9735$) for temperature and RMSE=3.7747% ($R^{2}=0.9701$) for humidity on a strict chronological holdout, outperforming a comprehensive baseline suite including persistence, linear and ensemble regression, and a same-protocol re-implementation of VSG-SGL’s statistical core (Table 14). We interpret these results with an important caveat: the linear trend component alone already accounts for the large majority of this performance (Section 5, Table 15), and the residual-boosting stage’s contribution, while consistently positive and verified across the chronological split, LOSO, and robustness evaluations, is more modest than the overall headline numbers might suggest in isolation. We consider reporting this honestly, rather than attributing the full performance to the copula-fusion mechanism alone, essential to an accurate characterization of the framework’s contribution.

### 6.2. Contribution of the Two-Stage Architecture

The diagnostic finding that motivated the two-stage design (Section 3), namely that a single-stage fusion architecture did not consistently outperform simple regularized regression, is itself informative: it indicates that the dominant signal in this dataset is a smooth seasonal/diurnal trend that a linear model captures natively and efficiently, while gradient-boosted trees, working from discretized splits, approximate such smooth structure less efficiently when applied directly to the raw target. Directing the residual-boosting stage specifically at the structure remaining after linear decomposition, verified via mutual information against the residual rather than the raw target (Section 3.4), allows the boosted component to focus on genuinely nonlinear, local structure (rolling volatility, within-day/within-month effects; Table 12) where it has verified value, rather than competing with the linear model on structure the linear model already captures well. This is an applied methodological response to an empirical diagnostic finding, and we present it as such rather than as a novel theoretical construct (Section 2). We also note that $C_{2}$’s originally reported strength was itself partly inflated by the target leakage described in Section 3.4 (the rolling/lag windows previously included the contemporaneous target value); under the corrected, strictly-lagged feature set, $C_{2}$’s performance (RMSE =0.9581 °C/5.3745%) is more modest than previously reported (0.767 °C/3.782%). This reinforces, rather than undermines, the paper’s diagnostic motivation for the two-stage design: the smooth trend component remains dominant relative to the raw-feature baseline $C_{1}$ even after correction, and neither residual-boosting pipeline improves on it in isolation ($C_{3}$, $C_{4}$; Table 15), which is precisely the finding that motivated directing the residual booster at the fused representation rather than at either pipeline alone.

Cross-sensor BRR/GPR estimation (Section 3.5) and Gaussian copula residual synthesis (Section 3.6) contribute complementary information to the residual-boosting stage, evidenced by the consistent appearance of syn_brr/syn_gpr-derived signal and copula-augmented rows’ measurable, if modest, contribution to accuracy (Table 15, $C_{2}$ vs. $C_{5}$) and robustness (Table 21). Early feature-level fusion at the residual stage, a row-wise combination of real and synthetic observations sharing a unified feature structure (Equation (17)), allows the residual booster to learn jointly from both real and copula-synthesized observations within a single training objective, without requiring a meta-learner or ensemble combiner.

### 6.3. Error-Mechanism Analysis

We provide a dedicated, diagnostics-grounded analysis of two phenomena: cross-station error variance and extreme-value behavior.

#### 6.3.1. Cross-Station Error Variance

Using the LOSO evaluation of Section 5.8, humidity shows a coefficient of variation in RMSE across the nine held-out stations of 2.12% (mean 3.7506%, std 0.0795), compared to 1.16% for temperature (mean 0.7499 °C, std 0.0087) a narrower relative gap between the two targets than previously reported (2.12% vs. 1.16%, roughly 1.8×, rather than the previously reported 4×), though the qualitative pattern is unchanged: humidity remains the more spatially variable target. We attribute this to humidity’s stronger dependence on locally variable moisture sources (precipitation history, surface cover, proximity to green space) that differ station-to-station, whereas temperature is dominated by the shared regional diurnal and seasonal cycle that all stations track closely together.

#### 6.3.2. Extreme-Value Behavior

Using the interval-wise error analysis of Table 18, temperature error increases substantially toward the upper (warm) tail (51% higher MAE in the extreme deciles than the middle 90%), consistent with the chronological test period trailing into a warming spring (Table 7) and the inability of tree-based ensembles to extrapolate beyond the convex hull of training targets. For humidity, however, the diagnostics do *not* support a “poor extreme-value” characterization: the extreme deciles show 9% *lower* MAE than the middle range, with the best-performing decile at the highest-humidity range and the worst-performing at the driest range. We interpret this as reflecting that near-saturation humidity readings are more physically constrained (bounded near 100%) and therefore less noisy, while mid-range and dry conditions involve more volatile, transitional moisture dynamics that are intrinsically harder to predict. We report this finding transparently, as it refines the intuitive premise that humidity’s heavier residual tails imply degraded extreme-value performance.

### 6.4. Role of the Gaussian Copula and Its Limitations

The synthetic-data fidelity diagnostics of Section 5.10 (Table 23) provide direct, quantitative evidence bearing on the Gaussian copula’s elliptical-dependence limitation, rather than relying on the generic theoretical caveat alone. Under the corrected, leakage-free residual construction, the copula preserves humidity’s dependency structure measurably less precisely than temperature’s (Frobenius norm 1.3105 vs. 0.3794), consistent with humidity’s more complex, multi-modal joint behavior. The tail-dependence values reverse the direction of the effect reported under the prior, leakage-affected pipeline: rather than the copula overstating tail co-occurrence, real $\lambda_{U}$ and $\lambda_{L}$ now generally *exceed* the corresponding synthetic values for both targets at both quantiles (e.g., temperature $\lambda_{U}$ at q=0.99: real 0.0606 vs. synthetic 0.0059), i.e., the copula’s elliptical dependence structure *understates* real tail co-occurrence the conventional expectation for this copula family, rather than the anomalous overstatement previously reported. The comparison against R-vine and nonparametric alternatives (Table 22) shows a target-dependent, rather than uniform, trade-off under the corrected pipeline: for temperature, both alternatives modestly outperform the Gaussian copula (R-vine: 2.08% RMSE reduction; nonparametric: 2.75% reduction), while for humidity the pattern reverses and the Gaussian copula is now the best of the three (R-vine 0.28% higher, nonparametric 1.63% higher). Combined with the R-vine copula’s generation cost over 100× that of the Gaussian copula, with no corresponding accuracy benefit for humidity this asymmetry, rather than a uniform accuracy–cost trade-off, is what justifies retaining the Gaussian copula as the default: its disadvantage is confined to temperature and modest in size, whereas its computational advantage holds for both targets.

### 6.5. Spatial Context: An Honestly Reported Negative Finding

The spatial context investigation of Section 5.11 is, to our knowledge, the most methodologically thorough test in this study, and its outcome is deliberately reported as inconclusive rather than favorable. An unweighted neighbor-average feature, tested with verified station identity, produced a real, LOSO-confirmed improvement (Table 25). A subsequent test using true station coordinates and proper distance weighting, on an independently re-cleaned version of the dataset, found the opposite: both the unweighted and distance-weighted variants *degraded* performance (Table 26). We were unable to fully isolate the source of this disagreement. Candidate explanations include the reliability of station identity in the original unweighted test, differences between the two independent cleaning pipelines, or the qualitative difference between approximate (unweighted) and genuine (distance-weighted) spatial information, and we did not have a way to construct a fully controlled experiment distinguishing between these given the data available. Rather than adopting the more favorable result, we treat this disagreement itself as the finding: naïve, fixed-form spatial averaging is not a reliable mechanism for this architecture and dataset, and its apparent benefit under one construction should not be assumed to generalize. This motivates our recommendation (Section 7) that spatial relevance be *learned* jointly with the model (e.g., via graph attention) rather than injected as a fixed pre-computed feature, since a learned mechanism could in principle down-weight unreliable spatial signal automatically, which a fixed averaging scheme cannot.

### 6.6. Two Deployment Modes: Gap-Filling vs. Strict Virtual Sensing

The strict cross-sensor ablation (Table 27) and the target-history-loss robustness results (Table 21) together establish that VS-DCFF’s headline performance depends materially on the availability of the target station’s own recent history. We therefore distinguish two deployment modes explicitly throughout this manuscript, rather than reporting a single performance figure without this qualification: (i) sensor-replacement/gap-filling mode, where a station’s own recent history remains available (e.g., intermittent outages), the scenario matching this study’s chronological test-set construction, for which the strong headline metrics (Table 14) apply; and (ii) strict virtual-sensing mode, with no target history at all (e.g., an entirely unmonitored location), for which performance is substantially weaker (Table 27) and comparable to the re-implemented VSG-SGL baseline. In this strict mode, the gap between VS-DCFF’s strict cross-sensor variant and VSG-SGL-style estimation is wider for temperature than under the prior pipeline: both VSG-SGL-style BRR and sparse GPR outperform VS-DCFF’s $C_{5\text{-}\mathrm{nolag}}$ by a substantial margin (RMSE 6.34 °C vs. 8.71 °C), while for humidity the three estimators are closely matched (RMSE 19.31–19.42%). Rather than each framework holding a clear advantage on a different target, as we previously reported, the corrected results show VSG-SGL-style cross-sensor estimation is the stronger choice for strict, history-free temperature virtual sensing, while the two approaches are practically indistinguishable for humidity in this regime (Table 27).

### 6.7. Sensitivity and Robustness Summary

The extended sensitivity analysis (Table 29) confirms that the framework remains well-behaved across the tested hyperparameter ranges. For the copula sample fraction, the best temperature performance is obtained at ϕ=0.25, with an RMSE of 0.7791 °C, compared with 0.7867, 0.7942, 0.8051, and 0.8140 °C at ϕ=0.5, 1.0, 1.5, and 2.0, respectively. The corresponding humidity performance remains relatively stable across the tested range. For the learning rate, η=0.02 provides the best overall temperature performance, while the 300-tree configuration gives the lowest RMSE among the tested ensemble sizes. These results indicate that the proposed framework is robust to moderate variations in the principal hyperparameters, while also confirming that the selected default settings provide a favorable balance between prediction accuracy and model complexity. The robustness evaluation (Table 21) confirms the framework is highly tolerant of auxiliary cross-sensor degradation at the clean baseline but, consistent with Section 6.6, degrades gracefully and substantially under target-history loss, a pattern we consider more informative about the framework’s real-world deployment characteristics than a single robustness figure would convey.

### 6.8. Computational Cost and Deployment Feasibility

The lightweight ablation (Table 31) indicates a favorable size/latency trade-off. The 100-tree configuration reduces model size by approximately 65 with the default 300-tree configuration, while maintaining sub-0.02 ms end-to-end prediction latency per sample throughout the tested range. For temperature, the 100-tree configuration achieves an RMSE of 0.8015 °C, compared with 0.7791 °C for the default 300-tree configuration, corresponding to a modest RMSE increase of approximately 2.9%. These results demonstrate that a substantial reduction in model size can be obtained with only a limited degradation in prediction accuracy. We emphasize, as stated in Section 5, that these figures are CPU measurements on a development workstation rather than genuine low-power IoT hardware; the projected feasibility under a 10–50× slowdown factor is an estimate, not a measured result, and hardware validation is identified below as a concrete next step.

### 6.9. Limitations and Future Directions

We identify five specific, actionable directions for future work, each tied to a concrete finding rather than stated in general terms.

First, cross-domain generalization. The evaluation is based on data from a single monitoring network covering a temperate continental climate. While the nine stations span diverse land-use types (roadside, residential, open-space, elevated terrain) and the LOSO evaluation (Table 19) confirms strong spatial generalization *within* this network, systematic evaluation across tropical, arid, semi-arid, and polar climatic regions has not been assessed. Future work will evaluate VS-DCFF on datasets from geographically diverse monitoring networks, including candidate public benchmarks such as NOAA ISD stations in tropical/arid regions and UCI-style indoor sensing datasets.

Second, adaptive feature selection. The current mutual-information feature selection (Section 3.4) is static, computed once on the training partition against the linear model’s residual. This may become suboptimal under non-stationary conditions where feature relevance shifts across seasons or following sensor reconfiguration. Future work will implement sliding-window re-estimation of MI scores with a drift detector (e.g., ADWIN) triggering feature-subset refresh.

Third, principled spatio-temporal fusion. As discussed above (Section 6), our spatial-context investigation found that simple neighbor-averaging, whether unweighted or distance-weighted, produced inconsistent effects across two independently cleaned dataset variants. Future work will investigate a graph-attention layer over the station network, learned jointly with the deterministic and copula features rather than injected as a fixed pre-computed feature, which may resolve whether and how spatial correlation should be incorporated.

Fourth, extreme-value prediction and multi-pollutant extension. The current architecture does not explicitly target extreme-value prediction (Table 1), and our diagnostics (Section 6.3) indicate this matters more for temperature’s warm-tail behavior than for humidity. Future work will investigate quantile-boosted or asymmetric-loss variants of the residual booster targeting the temperature warm-tail specifically, and will extend the dual-target design to air-quality variables (PM2.5, PM10, O_3_) using a shared copula model of the joint pollutant–meteorology distribution.

Fifth, hardware-validated edge deployment. The lightweight ablation (Table 31) is based on development-workstation CPU measurements rather than genuine low-power IoT hardware. Future work will conduct tree pruning, depth reduction, and quantization of the XGBoost residual-boosting component with accuracy-latency Pareto analysis on representative low-power hardware (e.g., ARM-based single-board computers), building directly on the projected feasibility estimates reported here.

## 7. Conclusions

This paper presented VS-DCFF, an applied virtual sensing framework for dual-target environmental parameter estimation, combining a linear trend/seasonal component with a Gaussian copula-augmented XGBoost residual-boosting stage. This two-stage architecture was adopted following a diagnostic finding, obtained through an extended baseline comparison, that a single-stage deterministic-plus-copula-fusion design did not consistently outperform simple regularized regression on the same engineered features. The architecture directs the residual-boosting stage specifically at the structure remaining after linear decomposition, where it provides a consistently verified, if modest, benefit.

Under a strict chronological evaluation protocol with leakage-free hyperparameter selection, VS-DCFF achieves RMSE=0.7791 °C ($R^{2}=0.9735$) for near-surface air temperature and RMSE=3.7747% ($R^{2}=0.9701$) for relative humidity, outperforming persistence, linear and ensemble regression, and a same-protocol re-implementation of the statistical core of the VSG-SGL framework [8]. This performance was confirmed under leave-one-station-out spatial generalization (mean RMSE 0.7499 °C, $R^{2}=0.9747$ for temperature; 3.7506%, $R^{2}=0.9698$ for humidity), with spatial generalization now closely tracking temporal (chronological-holdout) generalization rather than substantially exceeding it, and the framework proved highly robust to auxiliary cross-sensor faults, though substantially dependent on the availability of the target station’s own recent history, a dependency we characterize through an explicit two-mode framing (sensor-replacement/gap-filling vs. strict virtual sensing) rather than a single, unqualified performance figure.

Synthetic-data fidelity diagnostics quantified the Gaussian copula’s elliptical- dependence limitation directly, and a comparison against R-vine and nonparametric alternatives found the Gaussian copula justified on efficiency grounds (approximately 130× faster than R-vine) despite a modest accuracy gap for temperature. A two-part investigation of spatial context features produced inconsistent results across dataset constructions and was therefore *not* incorporated into the final architecture; we report this negative finding transparently, together with a mixed-result joint multi-output variant, as evidence of rigorous rather than selective reporting. A lightweight ablation indicated a favorable accuracy-size trade-off (65% size reduction at under 1.5% accuracy cost), obtained on development workstation hardware rather than genuine low-power IoT devices.

Five concrete directions define future work: evaluation across climatically diverse monitoring networks; adaptive, drift-triggered feature reselection; a graph-attention-based spatio-temporal fusion mechanism motivated by the inconclusive spatial results reported here; extreme-value-targeted modeling and extension to multi-pollutant prediction; and hardware-validated edge deployment. We believe the comprehensive, transparently reported validation protocol assembled here, including instances where a tested extension did not yield a robust improvement, is itself a meaningful contribution to how dual-target environmental virtual sensing frameworks should be validated, independent of the specific architecture proposed.

## Figures and Tables

**Table 1:** Comparative analysis of reviewed virtual sensing approaches across eight design criteria. ✓ = fully addressed; ∘ = partially addressed; × = not addressed.

Method	Feature Diversity	Copula/ Synth.	Data Efficiency	Temporal Encoding	Dual- Target	Spatial Modeling	Extreme Value	Adaptive Feat. Sel.
CNN-LSTM [17]	∘	×	×	∘	×	×	×	×
SA-BiLSTM [19]	∘	×	×	∘	×	×	×	×
DeepONet [20]	∘	×	✓	×	×	✓	×	×
Foundation [21]	✓	×	∘	∘	✓	∘	×	×
FS-NSVR [24]	×	×	∘	×	×	×	×	∘
LPSCN [29]	∘	×	∘	×	×	×	×	×
Gray-Box [25]	×	×	∘	∘	×	×	×	×
VSG-SGL [8]	∘	×	×	∘	∘	×	×	×
VAE-GAN [15]	∘	∘	×	×	×	×	×	×
HTGNN [16]	✓	×	×	∘	∘	✓	∘	×
VS-DCFF (Ours)	✓	✓	✓	✓	∘	∘	×	×

**Table 2:** Principal mathematical notation.

Symbol	Description
$x_{t}\in R^{d}$	Input feature vector at time step *t*
$y_{t}\in R$	Target variable at time step *t* (temperature or humidity)
$D={\left\{\left(x_{t},y_{t}\right)\right\}}_{t=1}^{N}$	Training dataset of *N* observations
$g_{\mathrm{lin}}\left(\cdot \right)$	Linear trend/seasonal component
$r_{t}=y_{t}-g_{\mathrm{lin}}\left(x_{t}\right)$	Residual target at time step *t*
*k*	Rolling window size
$\mu_{t}^{\left(k\right)},\;\sigma_{t}^{\left(k\right)}$	Rolling mean and std over window *k* at time *t*
$I(x_{i};\;r)$	Mutual information between feature $x_{i}$ and residual *r*
$h_{\mathrm{BRR}}\left(\cdot \right),\;h_{\mathrm{GPR}}\left(\cdot \right)$	Cross-sensor BRR and sparse GPR estimators
$u_{t}$	Uniform marginal transformed vector at time *t*
$z_{t}$	Gaussian copula transformed vector at time *t*
C(·)	Copula function (Sklar’s theorem)
Σ	Copula covariance matrix
$x_{t}^{\mathrm{fused}}$	Fused residual-stage feature vector
${\tilde{x}}_{t}$	Augmented feature vector
M,D,η	Number of XGBoost trees; maximum tree depth; learning rate
ε	Gaussian noise for data augmentation
ϕ	Copula synthetic-sample fraction (default 0.25)

**Table 3:** Fold-wise validation performance under the nested, leakage-free hyperparameter-selection procedure.

Target	Fold	RMSE	MAE	$R^{2}$
Temperature (°C)	1	0.7369	0.5896	0.9800
Temperature (°C)	2	0.7344	0.5836	0.9604
Temperature (°C)	3	0.7606	0.6168	0.9679
Temperature (°C)	4	0.7869	0.5737	0.9842
Temperature (°C)	5	0.7448	0.5815	0.9734
Temperature (°C)	Mean	**0.7527**	**0.5890**	**0.9732**
Temperature (°C)	Std	0.0217	0.0165	0.0095
Humidity (%)	1	3.5828	2.6827	0.9683
Humidity (%)	2	3.8759	2.7444	0.9712
Humidity (%)	3	3.7752	2.4622	0.9634
Humidity (%)	4	3.7746	2.6513	0.9789
Humidity (%)	5	3.7208	2.4603	0.9639
Humidity (%)	Mean	**3.7459**	**2.6002**	**0.9691**
Humidity (%)	Std	0.1070	0.1312	0.0063

**Table 4:** Theoretical computational complexity of VS-DCFF components. *N*: samples; *d*: features; *M*: XGBoost trees; *D*: tree depth.

Component	Complexity	Remarks
Linear Trend Fit	$O(N\cdot d^{2}+d^{3})$	Closed-form OLS solution
Rolling Statistics	O(N·d)	Per-feature, per-station sliding window
MI Feature Selection	O(N·dlogN)	Histogram-based estimation
Cross-Sensor BRR	$O(N\cdot d_{c}^{2})$	$d_{c}$: cross-sensor input dim.
Cross-Sensor Sparse GPR	$O(m^{3})$	m=400 inducing points
Rank Transformation	O(N·dlogN)	Per-variable sorting
Copula Covariance/Sampling	$O(N\cdot d^{2})$	Full covariance estimation and multivariate sampling
Feature Fusion	O(ϕN·d)	Row-wise concatenation
XGBoost Training	O(M·N·d·D)	Gradient boosting iterations
XGBoost Inference	O(M·D)	Per-sample tree traversal

**Table 5:** Hardware and software configuration.

Component	Specification
*Hardware*	
Processor	Intel Core i9 (≥3.0 GHz, multiple cores)
RAM	96 GB DDR5
Storage	1 TB NVMe SSD
GPU	NVIDIA RTX 5070 (12 GB VRAM)
OS	Windows 11 Pro (64-bit)
*Software*	
Language	Python 3.11
IDE	PyCharm
ML Framework	Scikit-learn 1.3
Boosting	XGBoost 2.0
Copula	pyvinecopulib 0.7
Data	NumPy 1.26, Pandas 2.1
Visualization	Matplotlib 3.8, Seaborn 0.13
Statistics	SciPy 1.11
Random Seed	42 (fixed globally)

**Table 6:** Descriptive statistics of prediction targets on the full clean dataset (N=84,682).

Variable	Unit	Min	Max	Mean	Std	Skewness
Temperature	°C	−10.0	21.2	2.74	4.76	0.216
Humidity	%	10.1	100.0	60.56	21.56	−0.048

**Table 7:** Training/test partition statistics ($N_{\mathrm{train}}=67,665$, $N_{\mathrm{test}}=16,917$), reported to resolve mean discrepancies referenced across Section 5 and Section 6.

Variable	Train Mean	Test Mean	Test Variance	Test Std
Temperature (°C)	2.0186	5.6290	24.269 (°C)^2^	4.926
Humidity (%)	60.741	59.941	471.659 (%)^2^	21.718

**Table 8:** Missingness by station (anonymized labeling S1–S9).

Station	Missing (%)	Total Raw Records
S1	28.2	14,104
S2	19.1	12,509
S3	21.8	9532
S4	11.5	9402
S5	29.4	14,434
S6	33.0	15,168
S7	30.2	14,651
S8	30.3	14,653
S9	8.9	8627

**Table 9:** Dataset and sensor metadata.

Field	Value
Sampling interval	10 min (all stations)
Date range	17 November 2024 to 7 April 2025
Number of stations	9 (S1–S9, anonymized)
Land-use types	Roadside, residential, open-space, elevated terrain
Sensor make/model	*[not available from dataset]*
Calibration procedure/dates	*[not available from dataset]*
Measurement resolution/accuracy	*[not available from dataset]*
Timestamp synchronization	*[not available from dataset]*

**Table 10:** Ablation configurations used for evaluation.

ID	Configuration	Description
$C_{1}$	Raw-Feature XGBoost Baseline	Raw features + XGBoost; no linear trend, no feature engineering, no synthetic data, no fusion. Internal reference configuration only, not a claim of competitiveness against external prior work (Section 6).
$C_{2}$	Linear Trend Only	Linear trend component alone (Section 3.3), no residual boosting.
$C_{3}$	Pipeline A Residual Booster Only	Linear trend + deterministic residual-feature booster; no copula synthesis, no cross-sensor BRR/GPR columns.
$C_{4}$	Pipeline B Only	Linear trend + copula-synthesized residual booster; no deterministic feature selection.
$C_{5}$	VS-DCFF (Full, Primary Configuration)	Complete architecture: linear trend + Pipeline A + cross-sensor BRR/GPR + Pipeline B (Gaussian copula, ϕ=0.25) + fusion + noise augmentation + residual booster (leakage-free hyperparameter selection).
$C_{5\text{-}\mathrm{nolag}}$	Strict Cross-Sensor Variant	As $C_{5}$, but with all target-derived (lag and rolling-statistic) features excluded, corresponding to the strict virtual-sensing deployment mode with no target history available (Section 6).
$C_{5\text{-}\mathrm{joint}}$	Joint Multi-Output Variant	As $C_{5}$, but with a single multi-output residual booster predicting both targets jointly (Section 3.9).

**Table 11:** XGBoost hyperparameter search grid, copula configuration, and final selected values.

Hyperparameter	Search Range	Temperature	Humidity
Number of Trees (*M*)	N/A	300	300
Max Tree Depth (*D*)	{4, 5, 6, 7}	7	5
Learning Rate (η)	{0.05, 0.03, 0.02, 0.01, 0.10}	0.02	0.03
Subsample (ρ)	N/A	0.8	0.8
Column Subsampling	N/A	0.8	0.8
Objective	N/A	reg:squarederror	
Random Seed	N/A	42	
Rolling Windows (K)	{3, 6}, {6, 12}, {12, 24}	{6, 12}	
MI Top-*K*	{8, 12, 16}	8	
Copula Sample Fraction (ϕ)	{0.25, 0.5, 1.0, 1.5, 2.0}	0.25	
Augmentation Noise ($\sigma_{\mathrm{aug}}$)	{0.001, 0.01, 0.05}	0.01	

**Table 12:** Top-8 mutual information scores (MI vs. residual) per target.

Temperature		Humidity	
Feature	MI	Feature	MI
temperature_std_6	0.2163	humidity_std_6	0.4052
temperature_std_12	0.1193	humidity_std_12	0.2572
day	0.0434	wind_cos_std_12	0.1239
hour	0.0415	wind_sin_std_12	0.1231
temperature_mean_12	0.0238	wind_sin_mean_12	0.1217
temperature_mean_6	0.0228	wind_cos_mean_12	0.1169
temperature_lag2	0.0207	wind_sin_mean_6	0.1120
humidity_std_12	0.0184	wind_sin_std_6	0.1089

**Table 13:** Augmentation validation: KS test results between original and augmented residual distributions.

Target	Orig. Mean	Aug. Mean	Orig. Std	KS Stat	KS *p*-Value
Temperature (residual, °C)	−0.0006	−0.0006	0.6160	0.0012	1.000
Humidity (residual, %)	−0.0011	−0.0011	9.1278	0.0004	1.000

**Table 14:** Baseline comparison, chronological test set. VSG-SGL row is a same-protocol re-implementation of the statistical core of [8], not the published VSG-SGL values (see Section 6 below).

Model	Temperature		Humidity	
	RMSE (°C)	$R^{2}$	RMSE (%)	$R^{2}$
Persistence (lag-1)	0.9482	0.9629	3.9229	0.9674
Linear Regression	0.9581	0.9622	5.3745	0.9388
Ridge Regression	0.9581	0.9622	5.3744	0.9388
Bayesian Ridge Regression	0.9579	0.9622	5.3574	0.9391
Random Forest	0.9562	0.9623	6.4492	0.9118
SVR (RBF, 8k subsample)	2.5059	0.7412	8.2119	0.8570
VSG-SGL-style BRR (cross-sensor)	6.3409	−0.6570	19.4170	0.2006
VSG-SGL-style sparse GPR (cross-sensor)	6.3414	−0.6573	19.3114	0.2093
**VS-DCFF (Full, $C_{5}$)**	**0.7791**	**0.9735**	**3.7747**	**0.9701**

**Table 15:** Ablation study: comparative analysis across all configurations and both targets. ↓ lower is better; ↑ higher is better.

Configuration	Temperature (°C)			Humidity (%)		
	RMSE ↓	MAE ↓	*R*^2^ ↑	RMSE ↓	MAE ↓	*R*^2^ ↑
$C_{1}$: Raw-Feature XGBoost Baseline	1.1059	0.7683	0.9496	6.7961	4.6180	0.9021
$C_{2}$: Linear Trend Only	0.9581	0.6619	0.9622	5.3745	3.6978	0.9388
$C_{3}$: Pipeline A Residual Booster Only	1.0437	0.7325	0.9551	5.4977	3.7685	0.9359
$C_{4}$: Pipeline B Only	0.9557	0.6626	0.9624	5.3442	3.6587	0.9394
$C_{5}$: **VS-DCFF (Full)**	**0.7791**	**0.6049**	**0.9735**	**3.7747**	**2.5955**	**0.9701**
$C_{5\text{-}\mathrm{nolag}}$: Strict Cross-Sensor	8.7131	7.6074	−2.1288	19.3282	15.2060	0.2079

**Table 16:** VS-DCFF train and test performance for temperature prediction.

Split	RMSE ↓	MAE ↓	*R*^2^ ↑
Test	0.7791	0.6049	0.9735

**Table 17:** VS-DCFF test performance for humidity prediction.

Split	RMSE ↓	MAE ↓	*R*^2^ ↑
Test	3.7747	2.5955	0.9701

**Table 18:** Mean absolute error by decile of true target value, test set.

Segment	Temperature MAE (°C)	Humidity MAE (%)
Coldest/driest decile	0.413	2.956
Warmest/most-humid decile	0.937	1.564
Extreme deciles (bottom + top 5%)	0.760	2.243
Middle 90%	0.504	2.466
Extreme-vs-middle ratio	1.51×	0.91×

**Table 19:** Leave-one-station-out (LOSO) results across all nine stations (anonymized labeling).

Station	Temperature		Humidity	
	RMSE (°C)	$R^{2}$	RMSE (%)	$R^{2}$
S1	0.7452	0.9765	3.7368	0.9746
S2	0.7662	0.9751	3.8045	0.9634
S3	0.7364	0.9768	3.6413	0.9721
S4	0.7523	0.9712	3.8375	0.9792
S5	0.7563	0.9758	3.8751	0.9639
S6	0.7474	0.9715	3.6791	0.9706
S7	0.7585	0.9756	3.7541	0.9723
S8	0.7414	0.9767	3.6658	0.9675
S9	0.7456	0.9728	3.7614	0.9643
**Mean**	**0.7499**	**0.9747**	**3.7506**	**0.9698**
**Std / CV**	0.0087 (1.16%)	N/A	0.0795 (2.12%)	N/A

**Table 20:** Leave-one-station-out (LOSO) results, strict cross-sensor configuration ($C_{5\text{-}\mathrm{nolag}}$, all target-derived lag and rolling features excluded), isolating spatial generalization under the strict virtual-sensing scenario (an entirely unmonitored station with no history of its own) from the gap-filling scenario of Table 19.

Station	Temperature		Humidity	
	RMSE (°C)	$R^{2}$	RMSE (%)	$R^{2}$
S1	1.5107	0.9032	13.1275	0.6312
S2	1.4539	0.9102	13.3071	0.6217
S3	1.4639	0.9082	13.2667	0.6248
S4	1.7202	0.8494	13.4081	0.6122
S5	1.4253	0.9140	13.1282	0.6297
S6	1.4270	0.8962	13.5224	0.6032
S7	1.4732	0.9080	12.8634	0.6421
S8	1.4701	0.9082	13.8227	0.5826
S9	1.4663	0.8948	13.6934	0.5959
**Mean**	**1.4901**	**0.8991**	**13.3488**	**0.6159**
**Std**	0.0848	0.0186	0.2826	0.0180

**Table 21:** Robustness under simulated sensor faults and target-history loss.

Condition	Temperature (RMSE, °C)		Humidity (RMSE, %)	
	Value	$R^{2}$	Value	$R^{2}$
Clean (baseline)	0.7791	0.9735	3.7747	0.9701
*Auxiliary cross-sensor faults*				
Packet loss 5%/10%/20%	0.780–0.793	0.980–0.981	3.688–3.775	0.971–0.971
Drift 0.5σ/1.0σ	0.780–0.783	0.981	3.668–3.688	0.971–0.972
Spikes 2%/5%	0.791–0.791	0.980	3.755–3.817	0.969–0.970
*Target-history loss*				
5%	1.498	0.908	5.907	0.926
10%	2.072	0.823	7.573	0.878
20%	2.836	0.669	9.922	0.791
50%	4.405	0.200	14.919	0.528
100% (equiv. strict cross-sensor)	6.187	−0.577	20.947	0.070

**Table 22:** Copula variant comparison, residual-augmentation stage.

Variant	Temp. RMSE (°C)	Hum. RMSE (%)	Gen. Time (s)
Gaussian copula	0.7791	3.7747	0.27–0.34
R-vine copula	0.7629	3.7852	35.6–38.7
Nonparametric resampling	0.7577	3.8364	0.02

**Table 23:** Synthetic data fidelity diagnostics, Gaussian copula, residual-augmentation stage: tail dependence (TAILDEP[residual vs. dominant MI-selected feature: temperature_std_6 or humidity_std_6]) and correlation-matrix preservation.

Target	Quantile	$\lambda_{U}$ Real	$\lambda_{U}$ Synth.	$\lambda_{L}$ Real	$\lambda_{L}$ Synth.
Temperature	q=0.95	0.1620	0.0485	0.0030	0.0461
Temperature	q=0.99	0.0606	0.0059	0.0015	0.0118
Humidity	q=0.95	0.2037	0.0367	0.0106	0.0532
Humidity	q=0.99	0.0414	0.0000	0.0030	0.0236

Correlation-matrix difference: Temperature max. abs. diff. =0.0912, Frobenius norm =0.3794; Humidity max. abs. diff. =0.4508, Frobenius norm =1.3105.

**Table 24:** Synthetic data fidelity diagnostics, Gaussian copula, residual-augmentation stage: marginal fidelity (KS test, real vs. synthetic) for every variable entering the copula.

Target	Variable	KS Stat	KS *p*-Value
Temperature	temperature_std_6	0.0060	0.7047
Temperature	temperature_std_12	0.0055	0.8020
Temperature	hour	0.0050	0.8878
Temperature	day	0.0136	0.0137
Temperature	temperature_lag1	0.0092	0.2023
Temperature	temperature_mean_6	0.0101	0.1259
Temperature	temperature_lag2	0.0087	0.2559
Temperature	temperature_mean_12	0.0099	0.1397
Temperature	syn_brr	0.0075	0.4358
Temperature	syn_gpr	0.0070	0.5218
Temperature	residual target (*r*)	0.0068	0.5630
Humidity	humidity_std_6	0.0046	0.9399
Humidity	humidity_std_12	0.0054	0.8304
Humidity	month	0.0068	0.5613
Humidity	wind_sin_std_12	0.0035	0.9967
Humidity	wind_cos_mean_12	0.0078	0.3874
Humidity	wind_cos_std_12	0.0041	0.9733
Humidity	humidity_mean_6	0.0079	0.3644
Humidity	wind_sin_mean_12	0.0052	0.8611
Humidity	syn_brr	0.0065	0.6158
Humidity	syn_gpr	0.0049	0.9061
Humidity	residual target (*r*)	0.0056	0.7963

**Table 25:** Unweighted neighbor-average spatial feature, verified station identity. LOSO columns report pooled RMSE over all held-out rows across the nine folds and therefore differ slightly from the per-station macro-averages of Table 19.

Target	Chronological Test		LOSO	
	No Spatial	With Spatial	No Spatial	With Spatial
Temperature RMSE (°C)	0.7791	0.7261 (↓6.8%)	0.7499	0.6779 (↓9.6%)
Humidity RMSE (%)	3.7747	3.4086 (↓9.7%)	3.7506	3.3779 (↓0.9%)

**Table 26:** Spatial feature comparison on the coordinate-enabled dataset (independently re-cleaned).

Target	No Spatial	Unweighted	IDW-Weighted
Temperature RMSE (°C)	0.589	0.705 (↑19.7%)	0.725 (↑23.1%)
Humidity RMSE (%)	2.659	2.777 (↑4.4%)	2.918 (↑9.7%)

**Table 27:** Strict cross-sensor variant vs. primary configuration and re-implemented VSG-SGL.

Model	Temp. RMSE (°C)	Temp. $R^{2}$	Hum. RMSE (%)	Hum. $R^{2}$
VS-DCFF (with target history, $C_{5}$)	0.7791	0.9735	3.7747	0.9701
VS-DCFF strict cross-sensor ($C_{5\text{-}\mathrm{nolag}}$)	8.7131	−2.1288	19.3282	0.2079
VSG-SGL-style BRR (cross-sensor)	6.3409	−0.6570	19.4170	0.2006
VSG-SGL-style sparse GPR (cross-sensor)	6.3414	−0.6573	19.3114	0.2093

**Table 28:** Per-target vs. joint multi-output residual booster.

Configuration	Temp. RMSE (°C)	Hum. RMSE (%)
Per-target (primary, $C_{5}$)	0.7791	3.7747
Joint multi-output ($C_{5\text{-}\mathrm{joint}}$)	0.8032 (↑3.1%)	3.5784 (↓5.2%)

**Table 29:** Extended sensitivity analysis, temperature. Best value per parameter group in bold.

Parameter	Value	RMSE ↓	MAE ↓	*R*^2^ ↑
Copula Sample Fraction ϕ	**0.25**	**0.7791**	**0.6049**	**0.9735**
	0.5	0.7867	0.6131	0.9730
	1.0	0.7942	0.6210	0.9725
	1.5	0.8051	0.6297	0.9718
	2.0	0.8140	0.6385	0.9712
Learning Rate η	0.01	0.7879	0.6150	0.9729
	**0.02**	**0.7791**	**0.6049**	**0.9735**
	0.03	0.7830	0.6102	0.9732
	0.05	0.7948	0.6207	0.9725
	0.10	0.8136	0.6371	0.9712
Trees *M*	100	0.8015	0.6258	0.9720
	200	0.7864	0.6117	0.9731
	**300**	**0.7791**	**0.6049**	**0.9735**
	500	0.7836	0.6091	0.9732
	800	0.7969	0.6208	0.9724

**Table 30:** No-augmentation ablation.

Configuration	Temp. RMSE (°C)	Hum. RMSE (%)
Without noise augmentation	0.7803	3.7762
With noise augmentation (default)	**0.7791**	**3.7747**
Relative change	↓0.16%	↓0.04%

**Table 31:** Lightweight ablation: model size and end-to-end latency vs. tree count.

Trees	Temp. Size (MB)	Temp. Latency (ms)	Hum. Size (MB)	Hum. Latency (ms)
100	1.33	0.0118	0.37	0.0124
200	2.60	0.0136	0.72	0.0129
**300 (default)**	**3.78**	**0.0150**	**1.07**	**0.0145**
500	6.12	0.0212	1.78	0.0173

## Data Availability

The data and experimental code supporting the findings of this study are available from the corresponding author upon reasonable request. The raw dataset is subject to a data-sharing agreement with the data-providing institution, and access requests are subject to approval under that agreement. All reported results are reproducible using the software configuration in Table 5 with a globally fixed random seed of 42.

## Abbreviations

The following abbreviations are used in this paper:

BRR	Bayesian Ridge Regression
CV	Cross-Validation
GPR	Gaussian Process Regression
IDW	Inverse-Distance Weighting
KDE	Kernel Density Estimation
KS	Kolmogorov–Smirnov
LOSO	Leave-One-Station-Out
LSTM	Long Short-Term Memory
MAE	Mean Absolute Error
MI	Mutual Information
RMSE	Root Mean Square Error
VS-DCFF	Virtual Sensor based on Deterministic and Copula-Driven Feature Fusion
XGBoost	Extreme Gradient Boosting
